# Defining the *Enterococcus faecalis* Fatty Acid Kinase System of Exogeneous Fatty Acid Utilization

**DOI:** 10.1111/mmi.70017

**Published:** 2025-08-02

**Authors:** Huijuan Dong, Qi Zou, John E. Cronan

**Affiliations:** ^1^ Department of Microbiology University of Illinois at Urbana‐Champaign Urbana Illinois USA; ^2^ Department of Biochemistry University of Illinois at Urbana‐Champaign Urbana Illinois USA

**Keywords:** Enterococcus, fatty acid, fatty acid kinase

## Abstract

Phospholipid synthesis in Firmicute bacteria differs markedly from that of the paradigm *Escherichia coli* pathway in that acyl phosphates are a key intermediate. Acyl phosphates are required for the first acylation step of the phospholipid synthesis pathway catalyzed by the PlsY acyltransferase and are synthesized by two different pathways. In the absence of exogenous fatty acids, *de novo* synthesized acyl‐acyl carrier protein (ACP) species are converted to acyl phosphates by the PlsX acyl‐ACP: phosphate acyltransferase, which transfers the acyl chain from ACP to inorganic phosphate. When exogenous fatty acids are present, these acids are converted to acyl phosphates by the FakAB fatty acid kinase and can be converted to acyl‐ACPs via PlsX. The active kinase is composed of the ATP‐requiring FakA subunit and a FakB fatty acid binding protein, which acts to present the fatty acid carboxyl group to the FakA kinase active site. In all Firmicutes examined to date, multiple FakB species are present. *Staphylococcus aureus* has two, whereas *Streptococcus pneumoniae* has three, whereas *Enterococcus faecalis* encodes four FakB proteins. We report the fatty acid preferences of these proteins obtained by use of mutant strains lacking each FakB or all possible combinations of three FakB deletions, plus a strain lacking all four FakB proteins. We also report the phenotype of a *∆fakA* strain and of a ∆*fakA* bypass suppressor mutant, plus the first indication of a role of the FakAB pathway in recycling of acyl chains.

## Graphical abstract

The enteric bacterium *Enterococcus faecalis* incorporates fatty acids from the medium and elongates any short chains to lengths suitable for membrane lipids. We have delineated the pathway.

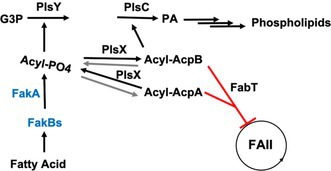

## 1 Introduction

**FIGURE 1:**
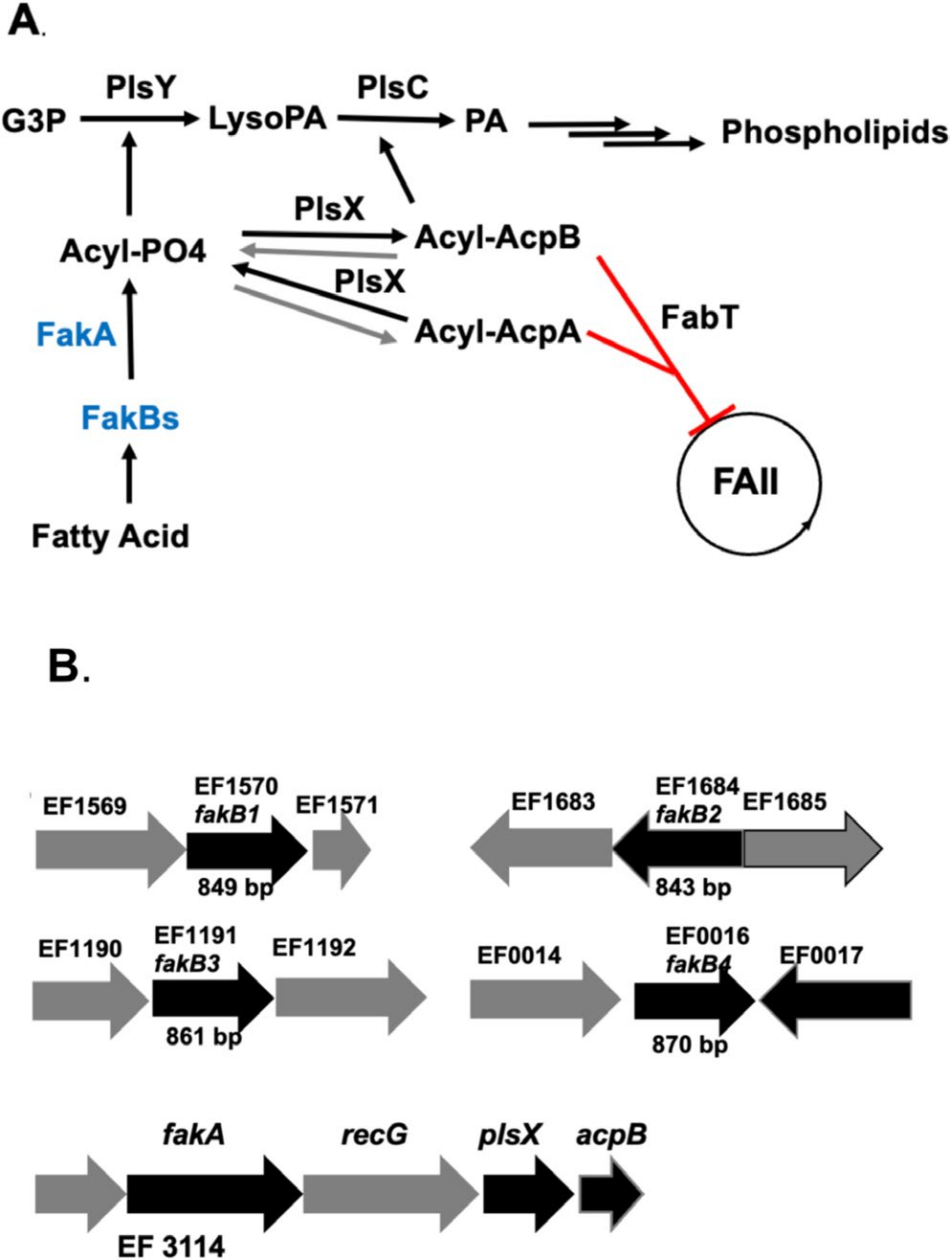
*E. faecalis* fatty acid uptake, synthesis and genome neighborhoods of the *fak* genes. (A) The pathway of exogenous fatty acid uptake, acylation, incorporation, and *de novo* synthesis regulation. Not shown are the ATP required for synthesis of acyl‐P by FakA and the phosphate required for PlsX synthesis of acyl‐P from acyl‐ACP species. (B) Genome neighborhoods of the four FakB genes and FakA.

Bacteria incorporate fatty acids from the environment by two distinct pathways (Yao and Rock 2017; Waters and Eijkelkamp 2024). In gram‐negative bacteria, fatty acids are internalized and trapped as acyl‐thioesters, which can be degraded or incorporated into complex lipids (e.g., phospholipids). In marked contrast, in the gram‐positive Firmicute bacteria, fatty acids are converted to acyl‐phosphate (acyl‐P) species, a direct precursor in phospholipid synthesis, or are converted into acylated derivatives of acyl carrier proteins (ACPs). Acyl‐ACPs can act as substrates in phospholipid synthesis, or if of short acyl chain length, can enter the fatty acid synthetic pathway for elongation to the chain lengths required for phospholipid synthesis (Zou et al. 2024). As discovered by Rock and coworkers, the key enzyme in this pathway is the FakA fatty acid kinase that phosphorylates the carboxyl groups of incoming fatty acids, producing acyl‐P species (Figure 1A) (Gullett et al. 2019; Lu et al. 2006; Parsons et al. 2014; Yao and Rock 2017) (Subramanian et al. 2022). However, FakA activity requires a second protein, called FakB, which binds the fatty acid and presents the fatty acid carboxyl to the FakA active site for phosphorylation by ATP (Parsons et al. 2014) (Subramanian et al. 2022). FakB proteins exchange bound fatty acids with fatty acids present in detergent micelles or liposomes (Yao and Rock 2017). Firmicute bacteria express a single FakA and multiple FakB proteins. *Staphylococcus aureus* has two, FakB1 and FakB2, that have different FA binding preferences, saturated and unsaturated acyl chains, respectively (Cuypers et al. 2019). *Streptococcus pneumoniae* has FakB1 and FakB2, whereas a third, FakB3, enables the utilization of the diunsaturated fatty acid, linoleic acid (Gullett et al. 2019). Four *fakB* genes have been identified in *Enterococcus faecalis* (Figures S2 and S3), *S. suis* , *S. pyogenes* , and *E. faecium* (Shi et al. 2022; Lambert et al. 2023; Zou et al. 2022; Waters and Eijkelkamp 2024).

The acyl‐P species produced by FakA‐FabB complexes are used by the PlsY acyltransferase for the synthesis of 1‐acyl‐*sn*‐glycerol‐3‐phosphate: the first acylation step of phospholipid synthesis (Yao and Rock 2017) (Figure 1A). The acyl‐P species can also be converted to acyl‐ACP species by the PlsX acyl‐ACP: phosphate acyltransferase. If the acyl chain is sufficiently long, the acyl‐ACPs are substrates for incorporation into position 2 of 1‐acylglycerol 3‐phosphate. Acyl‐ACP incorporation is catalyzed by the PlsC acyltransferase, resulting in the key phospholipid synthesis intermediate, phosphatidic acid (Yao and Rock 2017; Lu et al. 2006). Unlike *S. aureus* , *E. faecalis* has two ACP encoding genes, *acpA* and *acpB*. AcpA is required for *de novo* FA synthesis, whereas *acpB* is used only for extracellular FA incorporation (Dong and Cronan 2022; Zhu et al. 2019). In vitro, the freely reversible PlsX reaction prefers the transfer of the acyl‐phosphate acyl chain to AcpB rather than AcpA (Zou et al. 2022). In the absence of exogenous fatty acids, the *E. faecalis* acyl‐P substrate of PlsY is generated by PlsX catalyzed transfer of the acyl group of acyl‐AcpA to phosphate (Yao and Rock 2017; Zhu et al. 2019). Transcription of all *E. faecalis* fatty acid metabolism genes except *acpB* and *plsX* are regulated by the FabT repressor, which also autoregulates its own transcription. The acylated derivatives of AcpB (and more weakly—AcpA) act as ligands to enhance the binding of FabT to the fatty acid metabolism promoters to give down‐regulation of the fatty acid synthesis genes (Zhu et al. 2019; Zou et al. 2022).

We report that the *fakA* and *fakB* genes are essential for exogenous fatty acid incorporation in *E. faecalis* . Strains having deletions of *fakA* or of all four *fakB* genes (*∆fakB1∆fakB2∆fakB3∆fakB4*, abbreviated as *∆fakBquad*) are essentially completely defective in the ability to incorporate exogenous fatty acids into membrane lipids. FakA and the FakBs (as *∆fakBquad*) were also found to play a role in fatty acid recycling.

## 2 Results

### 2.1 The *fakA* and *fakB* Genes Are Not Essential for Growth of *E. faecalis*

The *fakA* genes of *Streptococcus pneumoniae and S. suis*, close relatives of *E. faecalis*, are reported to be essential, but based only on the inability to obtain gene disruptions (Shi et al. 2022; van Opijnen and Camilli 2012). However, we readily constructed both an *E. faecalis* ∆*fakA* strain and deletions of each of the four *fakB* genes (Figure S3). Moreover, strains having all combinations of triple *∆fakB* deletions and a quadruple (*∆quad*) strain lacking all four *fakB* genes were constructed by use of the CRISPr/Cas12a genome editing system (Chua and Collins 2022). The strains having single ∆*fakB* deletions were constructed by homologous recombination. Relative to the wild type strain, growth of the *∆fakA* and the *∆fakB quad* strains in M17 medium proceeded more slowly but achieved significant extents of growth, albeit less than that of the wild type strain, particularly with long incubation (Figure 3). Spontaneous faster‐growing suppressors of the *∆fakA* and *∆fakB quad* strains accumulated (see below). The *∆fakA and ∆fakB* strains showed a variety of growth phenotypes on M17 growth medium supplemented with diverse fatty acids (Figure S4). In some cases, a fatty acid inhibited growth. However, inhibition was seen only upon the long‐term growth of 2–3 days required for colony formation. Complementation with wild type *fakB* genes of the *∆fakB quad* strain for incorporation of [^14^C]‐labeled palmitate or stearate restored incorporation, albeit high‐level expression was required in some cases (Figure S5).

### 2.2 All Four *fakB* Genes Are Expressed and Produce Functional Proteins

**FIGURE 2:**
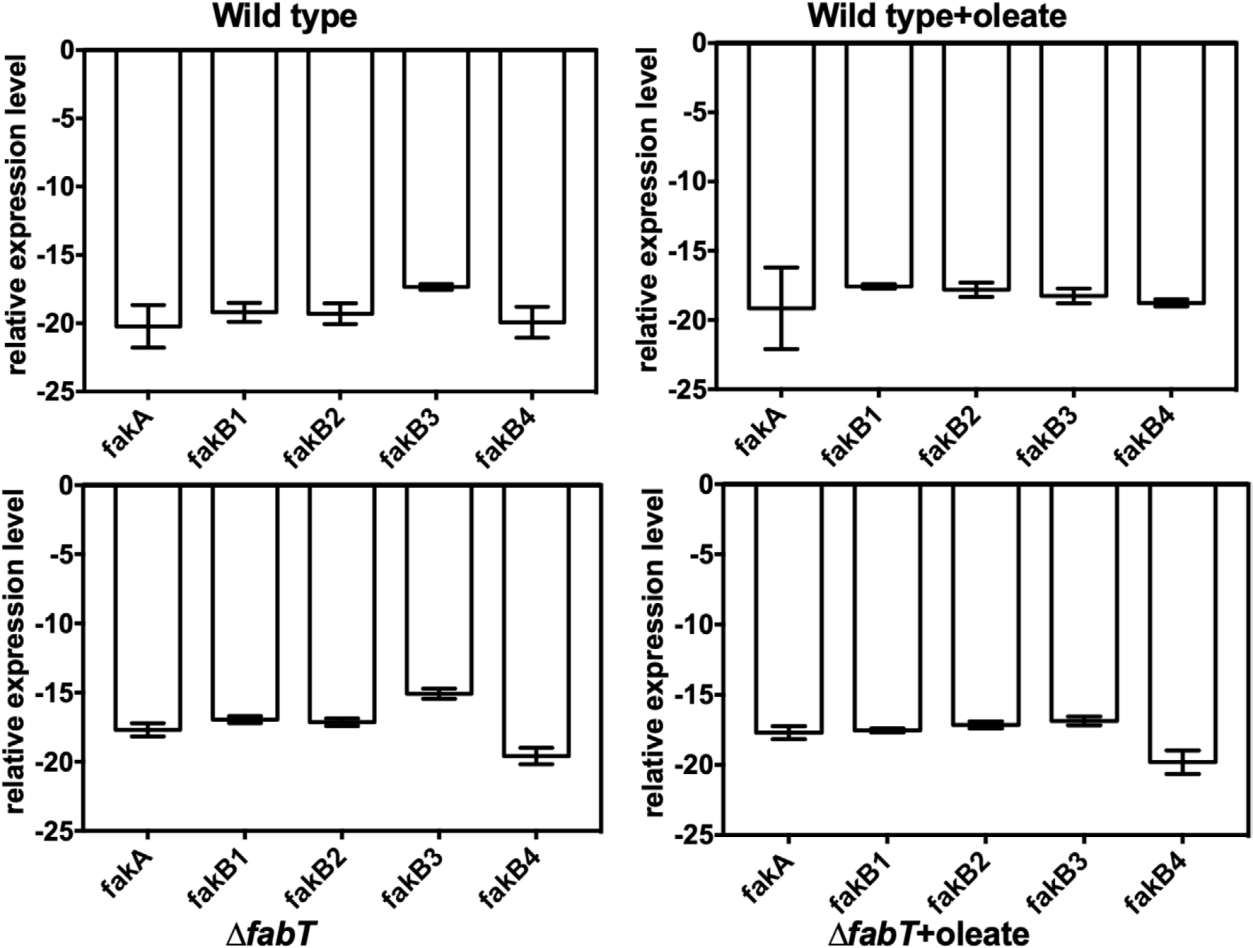
Expression of the *E. faecalis fak* genes assayed by RT‐PCR. The wild type (top row) and *∆fabT* (bottom row) strains were grown without or with oleic acid (100 μM) before RNA extraction for RT‐PCR analysis. The internal reference RNA was the 16S ribosomal RNA. This experiment was done in triplicate with the standard deviation shown by the error bars.

**FIGURE 3:**
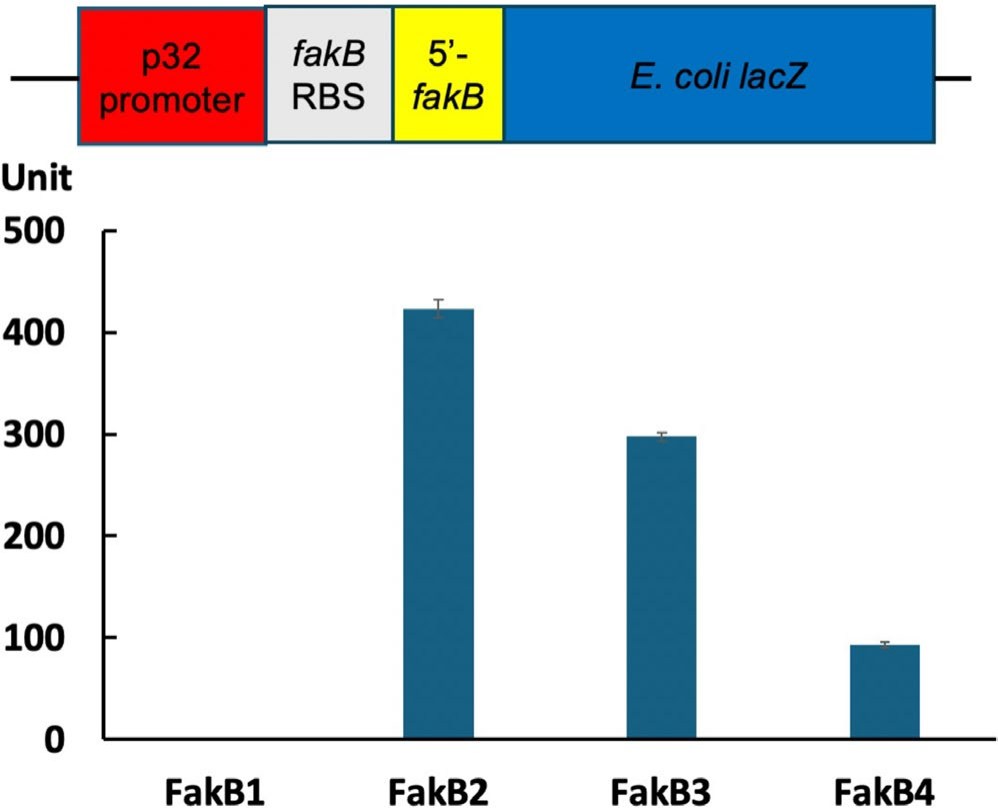
Translation of the *fakB* genes in *fakB‐lacZ* fusion constructs. The ribosome binding site together with the 5′ coding region for each *fakB* gene (−65 to +35 relative to the ATG initiation codon of each *fakB*) was fused to the promoterless *lacZ* gene of plasmid pBHK322 (Bi et al. 2014) and transformed into *E. faecalis* wild‐type strain. The transformed strains were assayed for β‐galactosidase activity. The P2 promoter plasmid is from *Lactococcus lactis* (Marelli and Magni 2010). This experiment was repeated twice in total and numerous times in unsuccessful attempts to detect activity of the FakB1 fusion. The error bars are ± SD.

**FIGURE 4:**
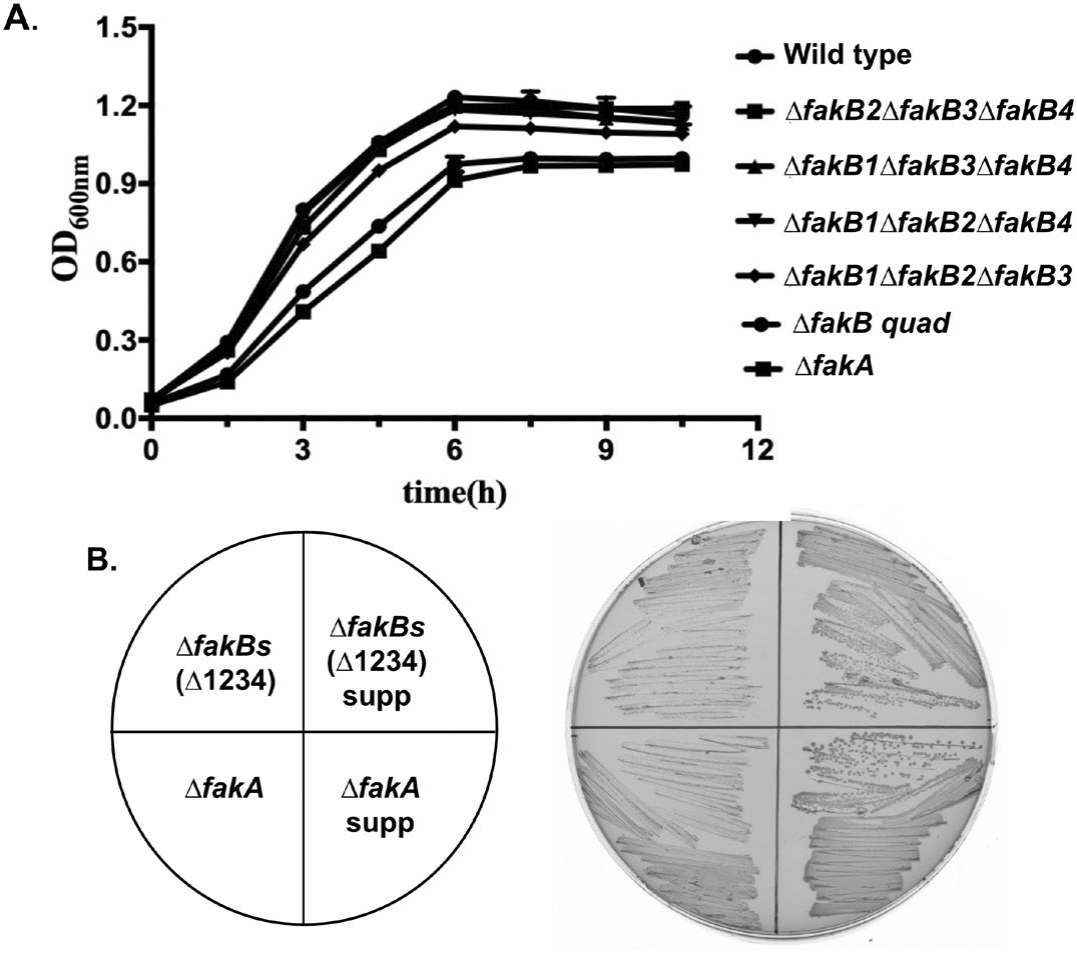
Growth of wild type and *fak* mutant strains, (A) Growth in M17 medium at 37°C. (B) Growth of the *∆fak* strains and the suppressor strains on M17 agar medium at 37°C. The growth experiments of Panel B were repeated several times for isolation of suppressor strains. The growth curves of Panel A were repeated twice but these could be compromised by suppressors that arise early in the growth of the cultures. Hence, Panel B provides better data because it is longer term, and suppressors cannot take over the cultures.

Although the Firmicutes studied to date encode only a single conserved FakA (Figure S1), all encode multiple FakB proteins, four in the case of *E. faecalis* (Figure S2). It is possible that some *E. faecalis* *fakB* genes may not be expressed at physiologically useful levels or at all. Another possibility is that some *fakB* genes may encode inactive proteins. To test expression, we assayed transcription of the four *fakB* genes and *fakA* by RT‐PCR (Figure 2). In the wild type strain, the five genes were expressed at similar levels. Lambert and coworkers reported that expression of *S. pyogenes fakB4* was regulated by FabT (Lambert et al. 2023). To ask if this is the case in the *E. faecalis fak* system, the expression levels in the wild type strain and a *∆fabT* strain lacking the FabT repressor were compared (Figure 2). Both strains were also grown in the presence of oleate. The transcription levels of the wild type and *∆fabT* strains were essentially the same for all five *fak* genes, and the expression levels in both strains were unaffected by growth with oleate (Figure 2). These data demonstrate that FabT does not regulate *E. faecalis fak* gene transcription. Since we had previously reported that *E. faecalis fabK*, a gene encoding a functional enoyl‐ACP reductase, is transcribed but not translated (Bi et al. 2014), we constructed translational fusions of the four FakB proteins to assay their efficiency of translation (Figure 3). The fusions to *E. coli* LacZ β‐galactosidase contained 65 bp upstream of the *fakB* ATG initiation codons plus 35 bp of the coding sequence. The longer upstream sequence was used since the ribosome binding sites of *E. faecalis* are not well defined. All four constructs gave blue colonies when transformed into a LacZ negative *E. coli* strain on X‐gal medium. Upon transformation into the *E. faecalis* wild‐type strain, three of the four constructs gave readily assayed β‐galactosidase (Figure 3). A perplexing result was that the FakB1 construct had no β‐galactosidase activity in *E. faecalis* despite its correct sequence and β‐galactosidase‐positive phenotype in *E. coli* . As shown below, the genetic constructs demonstrate that FakB1 is translated because the *fakB∆234* strain incorporated exogenous fatty acids, whereas the *fakB∆quad* strain did not. However, as also shown below, when assayed for the ability to facilitate attachment of acyl chains to AcpB in vitro, FakB had the lowest activity and most restricted substrate utilization of the four purified FakB proteins. This suggests that translation of FakB1 may be of little or no physiological consequence. Translation of *fakB2* was the most robust, followed by *fakB3* and *fakB4*, with translation of *fakB4* being about 20% that of *fakB2* (Figure 3). The mutant strains all grew (Figure 4) although the *∆fakA* strain and the strain lacking all four *fakB* genes grew slowly, which was more obvious in colony formation (Figure 4B).

**FIGURE 5:**
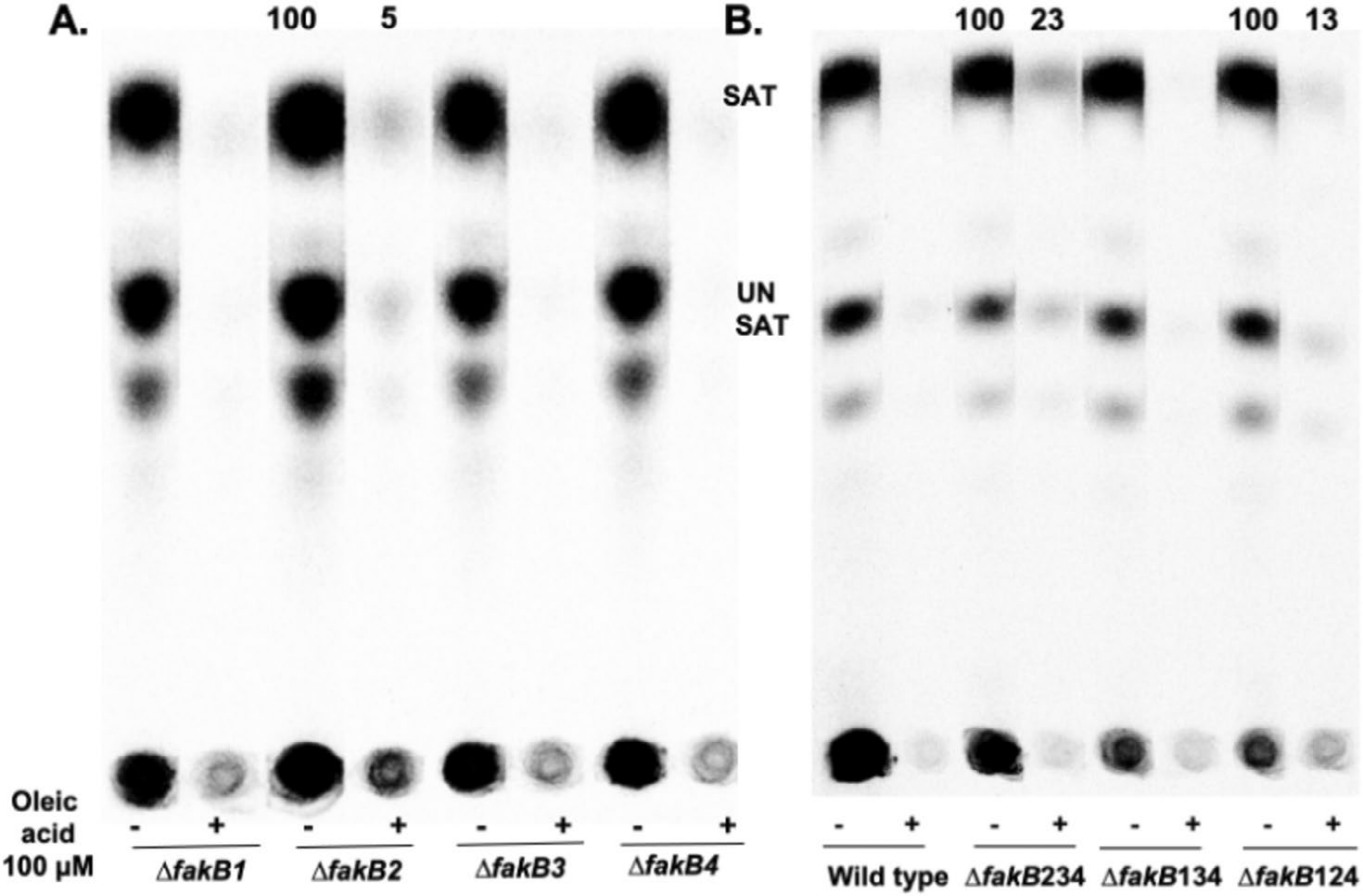
Abilities of *∆fakB* strains to repress *de novo* fatty acid synthesis. These [^14^C]acetate incorporation assays measure *de novo* fatty acid synthesis in the presence or absence of exogeneous 100 μM oleic acid. Conversion of oleic acid to oleoyl‐ACP requires a functional FakB which presents the oleate carboxyl to FakA resulting in oleoyl‐P. The oleoyl moiety is then transferred to AcpB (or less so to AcpA) by PlsX. Oleoyl‐ACP binds the FabT repressor and activates its binding to the fatty acid promoters resulting in repression of *de novo* fatty acid synthesis. (A) Single ∆fakB strains. (B) Strains with three of the four *fakB* genes deleted leaving only a single functional *fakB* gene. Regulation in the wild type strain is also given. Separation by argentation TLC and quantitation by phosphorimaging. The number above each lane gives the repression relative to that of the wild type strain. Where no number is given, no quantitative value could be obtained. Note that panels A and B are phosphoroimager scans of two separate plates (the Analtech plates are scored in fourths which allows smaller plates to be generated). The lines between the lanes were made prior to sample loading by scribing the coating of the TLC plate with a rake‐like tool. This results in ~1 mm of bare glass between 1 cm wide silica gel lanes. The bare glass prevents diffusion of compounds between adjacent lanes during development and also simplifies quantitation.

FakB function was first assessed by assaying the ability of an exogenous fatty acid to repress *de novo* fatty acid synthesis as measured by incorporation of [^14^C]acetate into phospholipid acyl chains (Figure 5). Four strains each lacking one of the four FakB proteins were tested for repression of *de novo* fatty acid synthesis by the addition of oleic acid. If a *∆fakB* mutant strain showed a lack of repression, this would indicate that the encoded FakB was required for both the FakA reaction and the subsequent PlsX reaction that converts oleic acid to the oleoyl‐ACP repression ligand. These results indicated that none of the four FakBs was specifically required for oleate activation (Figure 5A). A more definitive experiment tested repression activity in strains encoding only one FakB (three of the other four *fakB* genes were deleted). Each of these strains showed repression by oleic acid, although repression in strains lacking FakB1 was incomplete (Figure 5B). Repression indicated that each of the FakBs was active in fatty acid uptake and could support conversion to acyl‐ACP species via the FakA and PlsX reactions (Figure 1A).

### 2.3 Assays of FakB Activity and Selectivity

Given that all four *E. faecalis fakB* genes are expressed, why are four FakB proteins present, and are they fatty acid selective? We performed several assays for FakB function and fatty acid selectivity. Although the regulation of *de novo* fatty acid synthesis (Figure 5B) indicated that the FakBs are active, the assay requires several downstream steps, each of which may have acyl chain selectivity. The steps are conversion of the fatty acid to acyl‐P, transfer of the acyl chain to PlsX, which relays the acyl chain to AcpB or (less likely) AcpA, and binding of the acyl‐ACP to FabT (Figure 1A). A second assay used in prior work on the *S. aureus* FakBs was direct binding of radioactive fatty acids to purified FakB proteins (Parsons et al. 2014). We have performed similar assays (Table S1) but these assays give no information on the ability of a given FakB‐fatty acid complex to bind FakA for efficient presentation to the FakA active site, which can vary (Cuypers et al. 2019). Hence, binding to a FakB does not necessarily reflect in vivo activity. An example is that although FakB1 and FakB3 bind stearic acid (C18:0) tightly (Table S1), the acid was not efficiently passed through the FakA and PlsX reactions to AcpB in the coupled in vitro assays described below. Another shortcoming of these assays is that micelles formed by long chain fatty acids may bind nonspecifically to denatured proteins.

An in vivo assay involved feeding fatty acids to strains having deletions of three of the four *fakB* genes. This assay avoids the FabT interaction step and allowed both acyl‐P and acyl‐ACPs to provide acyl chains for phospholipid synthesis. We first assayed incorporation of oleate into phospholipids by GC–MS using a GC column (CP‐Si88) that completely resolves the methyl esters of oleate (C18∆9) and the native C18 monounsaturated acid, *cis*‐vaccenate (C18∆11). This allowed assay of Fak protein function by growth of the *fak* gene deletion strains with oleate and determination of oleate incorporation into phospholipids to provide a measure of Fak protein function (Table 1). The wild type *E. faecalis* FA2‐2 strain readily incorporated high levels of oleate into phospholipid. Indeed, oleoyl chains replaced all the native unsaturated chains and became the major phospholipid acyl chain (Table 1). The *∆fakB2∆fakB3∆fakB4* strain incorporated about half as much oleate as the wild type strain, indicating a role of *∆fakB1* in oleate utilization, whereas the *∆fakB1∆fakB3∆fakB4* and *∆fak1∆fakB2∆fakB4* strains had wild type oleate incorporation levels, showing that the FakB protein remaining in these strains efficiently utilized oleate (Table 1). The *∆fakB1∆fakB2∆fakB3* strain was the only triply deleted strain that failed to incorporate detectable oleate (Table 1). These data argue that *∆fakB4* plays a minor role in oleate incorporation in vivo. Both the *∆fakB quad* and ∆*fakA* strains failed to incorporate detectable oleate (Table 1).

**TABLE 1:** Fatty acid compositions of the wild type, *∆fakB*, *∆fakA*, and *∆fakA* suppressor strains.

Acyl chains	Wild type	Wild type+oleate	*∆fak2 ∆fak3 ∆fak4*	*∆fak2 ∆fak3 ∆fak4*+oleate	*∆fak1 ∆fak3 ∆fak4*	*∆fak1 ∆fak3 ∆fak4*+oleate	*∆fak1 ∆fak2 ∆fak4*	*∆fak1 ∆fak2 ∆fak4*+oleate	∆*fak1 ∆fak2 ∆fak3*	∆*fak1 ∆fak2 ∆fak3*+oleate	*∆fakB quad*	*∆fakB quad*+oleate	*∆fakA*	*∆fakA* supp	*∆fakA* supp+oleate
C14:0	5.3 ± 0.3	—	4.7 ± 0.2	—	4.4 ± 0.4	—	4.8 ± 0.4	—	5.7 ± 0.3	—	6.4 ± 0.7	—	2.9 ± 2.2	1.5 ± 0.5	0.5 ± 0.2
C16:0	36.4 ± 1.6	9.5 ± 0.8	46.6 ± 2.9	31.6 ± 2.9	43.1 ± 1.9	12.5 ± 1.0	38.7 ± 1.7	9.6 ± 1.1	44.4 ± 0.5	59.8 ± 0.1	44.0 ± 2.5	52.7 ± 3.4	39.7 ± 3.7	30.5 ± 0.4	26.3 ± 2.0
C18:0	6.6 ± 0.9	4.9 ± 1.2	5.5 ± 3.0	6.2 ± 1.2	6.7 ± 1.0	3.6 ± 0.3	4.6 ± 0.6	3.3 ± 0	2.3 ± 0.4	1.9 ± 1.9	3.9 ± 0.6	4.0 ± 5.6	6.4 ± 1.3	3.4 ± 1.0	11.9 ± 1.3
C19:0cyclo	7.7 ± 0.7	19.3 ± 0.9	7.2 ± 0.3	15.7 ± 1.1	3.8 ± 0.4	16.3 ± 0.9	7.8 ± 1.6	17.2 ± 1.4	8.4 ± 1.7	16.5 ± 2.1	11.4 ± 1.1	11.4 ± 8.1	12.2 ± 2.1	23.4 ± 0.8	9.3 ± 0.8
C14:1(7)	0.3 ± 0.2	—	0.2 ± 0	—	—	—	0.3 ± 0.1	—	0.3 ± 0	—	—	—	—	—	—
C16:1(9)	9.6 ± 0.7	—	5.4 ± 0.2	—	5.6 ± 0.9	—	8.4 ± 0.7	—	8.1 ± 0.8	—	8.5 ± 1.2	—	5.6 ± 0.6	6.1 ± 0.6	2.8 ± 0.3
C18:1(11)	34.1 ± 1.2	—	30.6 ± 0.6	10.1 ± 1.4	36.4 ± 2.2	2.5 ± 1.0	35.3 ± 1.0	6.4 ± 1.2	30.8 ± 1.4	21.8 ± 0.9	25.9 ± 2.5	32.0 ± 8.9	33.3 ± 4.3	35.2 ± 1.4	49.2 ± 2.6
C18:1(9)	—	67.3 ± 2.5	—	36.5 ± 1.4	—	65.2 ± 1.2	—	65.7 ± 1.9	—	—	—	—	—	—	—
USF/SFA	1.07	6.46	0.76	1.65	0.84	5.25	1.08	6.92	0.91	0.62	0.84	0.76	1.04	1.82	1.58

*Note:* The cultures were grown in M17 medium. Oleate (C18:1(∆9)), when added, was at 100 μM. UFA/SFA is the ratio of unsaturated to saturated acyl chain species. Note that addition of oleate decreases the level of the native *cis*‐vaccenate (C18:1(∆11)).

### 2.4 A Defined Assay for Conversion of Fatty Acids to Acyl‐AcpB Thioesters

**FIGURE 6:**
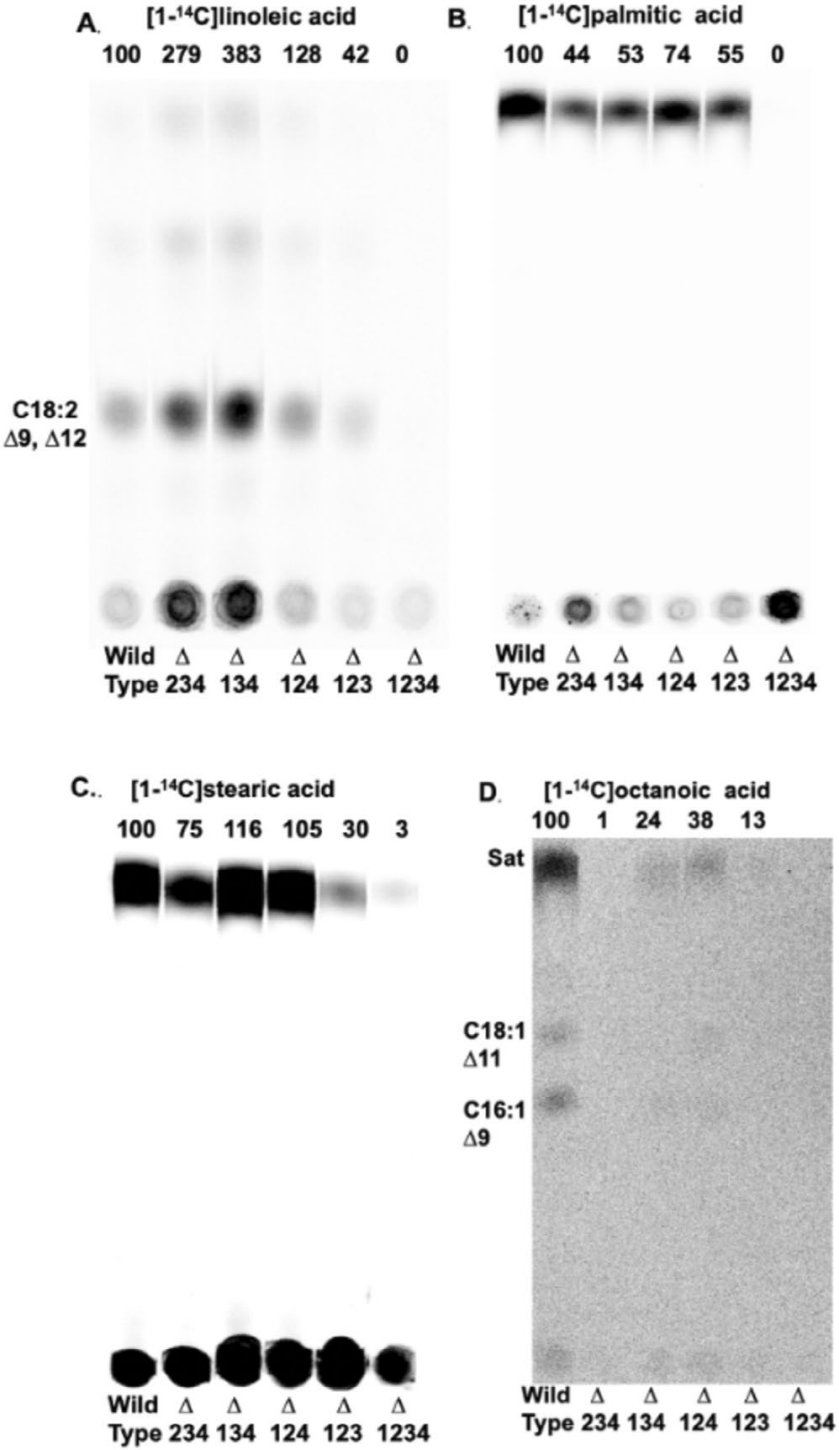
FakB activity in incorporation of [1‐^14^C]‐labeled fatty acids into phospholipids. (A) [1‐^14^C]linoleic acid (C18∆9,12) incorporation. (B) [1‐^14^C]stearic acid (C18) incorporation. (C) [1‐^14^C]palmitic acid (C16) incorporation. (D) [1‐^14^C]octanoic acid (C8) incorporation. Note that octanoic acid must be elongated to become a phospholipid acyl chain and is a precursor for both unsaturated and saturated fatty acids in *E. faecalis* (Zou et al. 2024). The number above each lane gives the incorporation relative to that of the wild type strain (quantitation by phosphorimaging). Argentation TLC separates by double bond number, position and configuration so the saturated acids, palmitate and stearate, run close to the solvent front due to lack of a double bond. The TLC plate of panel D was overexposed to allow detection of [1‐^14^C]octanoic acid into phospholipids which requires chain elongation in addition to uptake. Overexposure was responsible for the increased background. TLC plates A–D are separate scribed quarter plates as described in the legend to Figure 5. These are four separate TLC plates. The lines that separate the lanes were made prior to sample loading as given in Figure 5.

Fatty acid composition analysis was not a sensitive means to assay utilization of exogenous acyl chains that are native to *E. faecalis*. Hence, incorporation of various [1‐^14^C]‐labeled acids into membrane phospholipids was used (Figure 6). Note that *E. faecalis* and closely related bacteria lack a β‐oxidation pathway, so labeled acids are not degraded. The saturated fatty acids, stearate (C18) and palmitate (C16), and the diunsaturated linoleic acid showed some incorporation selectivity. All strains that retained a FakB had about half the [1‐^14^C]palmitate incorporation level of the wild‐type strain, suggesting that several FakBs function with this fatty acid (Figure 6B). This was also the case for [1‐^14^C]stearate although the strain lacking FakB4 showed significantly lower incorporation (Figure 6C). [1‐^14^C]Octanoate (C8) was poorly incorporated (Figure 6D). Octanoic acid is poorly bound by the FakB proteins (Table S1) and cannot be incorporated into phospholipids unless elongated to the long‐chain species required for phospholipid synthesis (Zou et al. 2024). The diunsaturated acid, linoleate, unexpectedly showed greater incorporation than the wild‐type strain in those strains that retained either FakB3 or FakB4 (Figure 6A). This suggests that in the wild‐type strain, there may be competition among FakBs for binding to FakA.

In these assays, the *E. faecalis* strains lacking all four FakB proteins (*fakB∆quad*) or FakA were almost completely defective in exogenous fatty acid incorporation into phospholipids (Table 1, Figure 6, Figure S5). This demonstrated that the *E. faecalis* strain FA2‐2 lacks other proteins having FakA or FakB activity. However, in some experiments, these strains showed low or trace levels of incorporation. This can be attributed to selection for bypass suppressors that accumulated during the growth of the cultures for labeling. The *∆fakB quad* and *∆fakA* strains grow relatively poorly on the labeling medium (Figure 4) thus providing a strong stochastic selection for faster‐growing suppressor strains.

**FIGURE 7:**
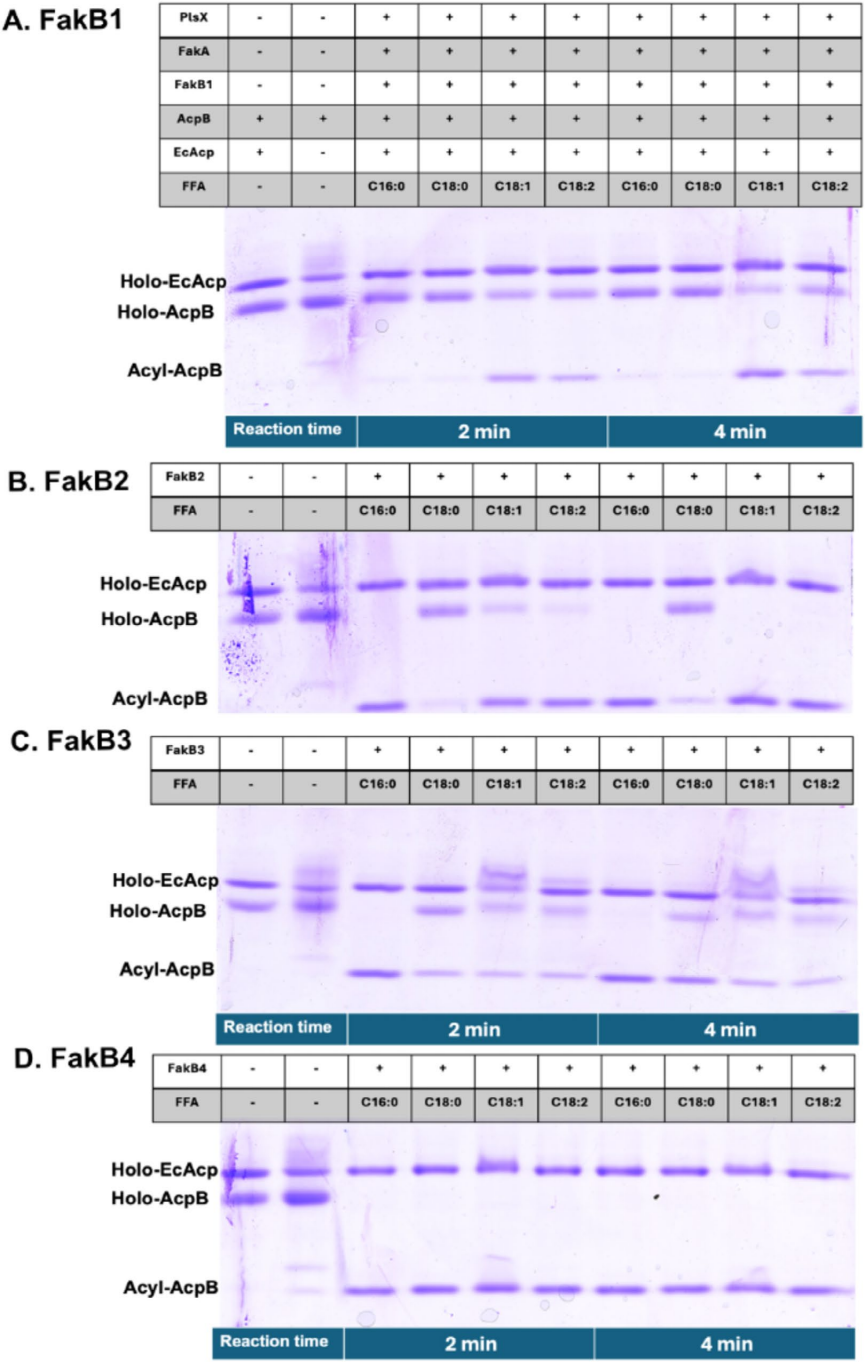
Function of each of the four FakB proteins in attachment of four different acyl chains to AcpB. In this in vitro assay the FabB binds the fatty acid and presents the fatty acid carboxyl group to FakA. FakA plus ATP converts the fatty acid to acyl‐P and PlsX then transfers the acyl chain from acyl‐P to Holo‐AcpB to form acyl‐AcpB. Samples were taken after 2 and 4 min of incubation. *E. coli* ACP (EcACP) which is not a substrate which copurified with AcpB was used as a loading control. Each of the proteins were in purified form (Figure S6). The concentration of each FakB was 0.17 μM and that of the fatty acids was 50 μM. The concentrations of FakA, PlsX, AcpB and ATP were 2, 2, 80, and 0.2 mM, respectively. The two left most lanes are the holo‐ACP standards. Note that bound fatty acids do not affect the properties of purified FakBs because fatty acids bound to purified FakBs exchange with the excess micellular fatty acids and are diluted out by mass action (Cuypers et al. 2019). Note that the 2 and 4 min incubations were done as separate experiments.

Although assays using oleate and radioactive fatty acids have the advantage of being performed in growing cultures, the exogenous fatty acids must compete with *de novo* synthesized acyl chains for incorporation into phospholipids. Also, the different FakBs could compete for binding to FakA, which could mask acyl chain selectivity of a FakB protein. To avoid these complications, an in vitro system of purified proteins (Figure S6) containing FakA, a single FakB, PlsX, and AcpB was used to assay the roles of the four FakBs in the incorporation pathway by production of acyl‐AcpB species (Figure 7). The system had obligate requirements for both FakA and a FakB and allowed a direct side‐byby‐side comparison of the four FakBs with a given fatty acid (Figure 7). We found that FakB1 functioned with the unsaturated acids but poorly with palmitate (C16) and stearate (C18) (Figure 7A). FakB2 functioned very well with the unsaturated acids and with palmitate but poorly with stearate (Figure 7B) whereas FakB3 preferred saturated fatty acids (Figure 7C). FakB4 showed complete conversion of all four tested fatty acids to acyl‐AcpB species even at the first time point (Figure 7D). Hence, in this assay, FakB4 seems the “best” overall FakB, with FakB2 in second place. FakB1 showed only modest activity and only with the unsaturated fatty acids (Figure 7A,C).

### 2.5 Loss of Regulation of Fatty Acid Biosynthesis Bypasses the Loss of FakA

**FIGURE 8:**
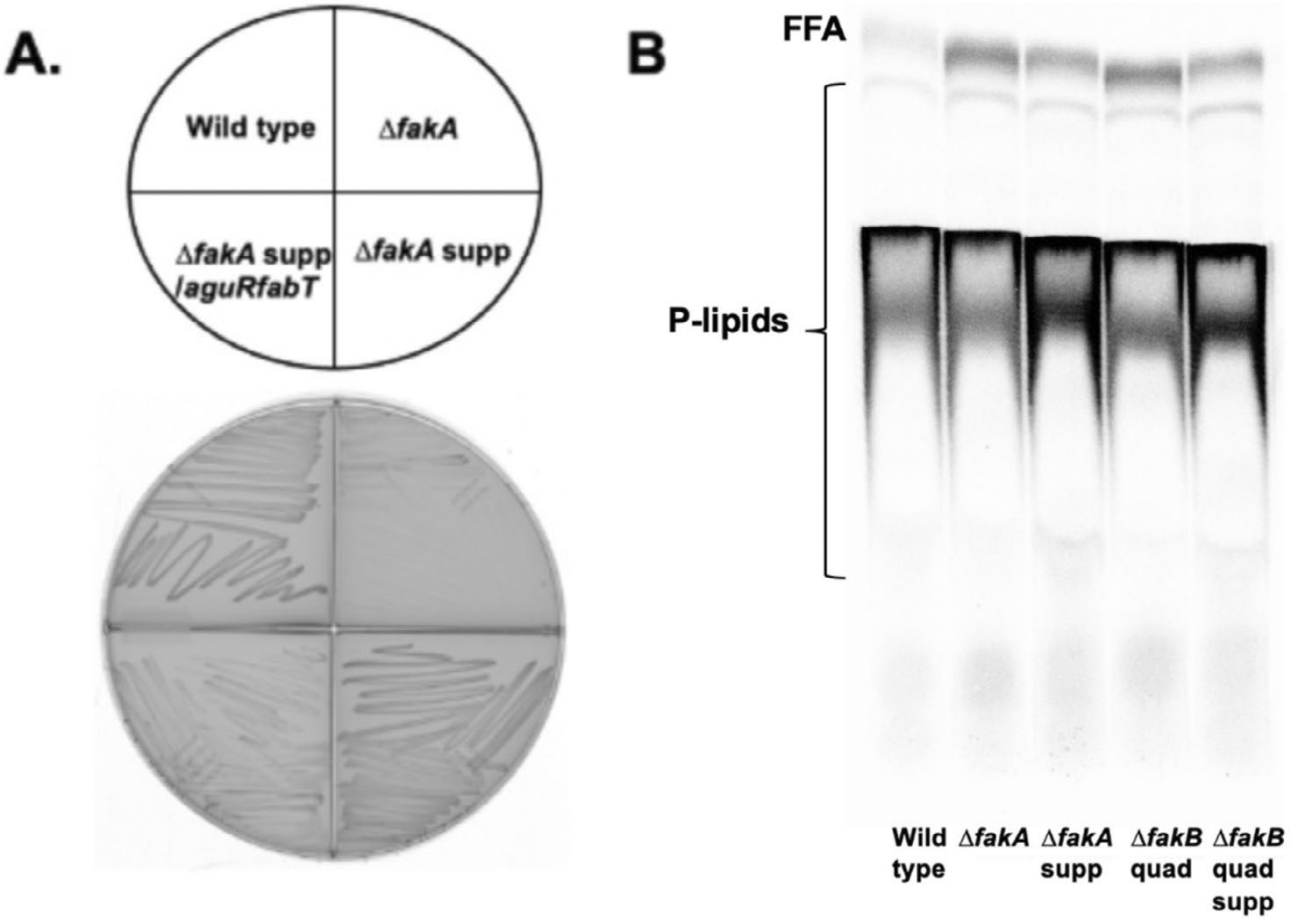
The FabT mutation of the *fakA* suppressor strain results in increased fatty acid synthesis and both FakA and FakBs are required for fatty acid recycling. (A) Growth of the wild type strain, the *∆fakA* strain and its suppressor (supp) strain. The *fabT* mutation of *∆fakA* suppressor strain was complemented with a plasmid encoding wild type FabT under control of the agmatine inducible promoter (Linares et al. 2014). (B) Accumulation of free fatty acids (FFA) in wild type, *∆fakA* and *∆fakB* strains and suppressor (supp) derivatives. Cultures were labeled with [1‐^14^C]acetate and then samples of the cultures (cells plus medium) were processed for lipid extraction and chromatography (Cho and Cronan Jr. 1995). The silica gel G TLC plate was developed twice. The first development was in hexanes/ether/acetic acid, (70:30:2) to the top of the plate to separate FFA from phospholipids and then a second development a second time with chloroform/methanol/acetic acid (65:25:8) was used to move the phospholipids off the origin. The second development was halted when the solvent reached about 75% of the plate height. The ratios of FFA radioactivity to phospholipid radioactivity are wild type, 0.028; *∆fakA*, 0.133, *∆fakA* supp, 0.058, *∆fakBquad* 0.137, and *∆fakBquad* supp 0.054. The TLC plate was scribed as described in Figure 5. This experiment was done several times although different TLC separations were used. This is a single TLC plate. The lines that separate the lanes were made prior to sample loading as given in Figure 5.

As shown in Figure 7B, the *∆fakA* strain accumulated suppressor mutants visible as large colonies on a bed of small colonies. Genome sequencing showed one such *∆fakA* suppressor strain contained a FabT point mutation in the residue 44 codon that resulted in a charge shift from glutamate to lysine. This (thus far) invariant FabT glutamate residue lies within a strongly conserved cluster of hydrophobic residues (Lambert et al. 2022). FabT is the repressor that regulates transcription of the fatty acid biosynthesis genes and autoregulates its own synthesis. When the *∆fakA* suppressor strain was complemented with wild type *fabT*, the complemented strain grew much more poorly than the suppressor mutant strain (Figure 8A). This indicated that the *fabT* suppressor mutation was a recessive, loss‐of‐function allele. This finding posits that the *∆fakA* suppressor strain would lack regulation of fatty acid synthesis and autoregulation of *fabT* expression. Indeed, when assayed by [1‐^14^C]acetate incorporation into phospholipid acyl chains, incorporation in the *∆fakA* suppressor strain was greater than that of the *∆fakA* strain (Figure 8B).

Note that we also observed accumulation of suppressors of the *∆fakB quad* strain that were significantly weaker than the *fabT* suppressor of the ∆*fakA* deletion. The mutation is a conservative substitution of hydrophobic residues, isoleucine for methionine, at residue 120 in FabF, a major 3‐ketoacyl‐ACP synthase. In the FabF crystal structure (PDB 1OX0) of the closest relative, *S. pneumoniae*, residue 120 may be involved in dimerization, although this is conjecture because the *S. pneumoniae* FabF protein (PDB 1OX0) is only ~60% identical to the *E. faecalis* FabF. Another factor in choosing not to further study *fakB∆quad* suppression was that genome sequencing of putative suppressor strains isolated on AC medium (richer in fatty acids than M17 medium) disclosed no mutations in these genomes.

### 2.6 Strains Lacking FakA or All Four FakBs Appear Deficient in Fatty Acid Recycling

Why do strains that lack FakA or all four FakBs grow poorly? This seems likely to be due to an inability to recapture any acyl chains liberated by thioesterase action and/or acyl‐P cleavage for new synthesis of new phospholipids. Indeed, about 5‐fold greater levels of free fatty acids were found in the medium of both *∆fakA* and *∆fakBquad* strains (Figure 8B). The suppressor strain had ratios intermediate between those of the wild type and their parent strains. The analysis method was designed to include any membrane vesicles (Afonina et al. 2021). Note that the use of the *E. faecalis* Fak pathway to recycle fatty acids parallels findings in Gram‐negative bacteria where the FadD acyl‐CoA synthase, an enzyme required for exogenous fatty acid uptake, is used to recycle *de novo* synthesized acyl chains released by hydrolysis (Dong et al. 2024; May and Silhavy 2018; Pech‐Canul et al. 2011). In these bacteria, recycling requires only FadD because, unlike the Firmicute acyltransferases, the acyltransferases of these bacteria can directly utilize acyl‐CoAs for phosphatidic acid synthesis (Yao and Rock 2017).

## 3 Discussion

Despite extensive efforts, we were unable to uncover a strong rationale for the presence of four *fakB* genes in *E. faecalis* . All four genes are expressed and produce active proteins, and some FakB proteins show fatty acid selectivity. In the in vitro acyl‐AcpB synthesis assay, the saturated long‐chain fatty acids palmitate (C16) and stearate (C18) are poor substrates for FakB1. FakB4 used both saturated acids, whereas FakB2 used palmitate (C16) but not stearate (C18). All four proteins used oleate (C18:1). All functioned with linoleate (C18:2) although FakB2 was the most efficient with this acid and FakB3 functioned weakly with both unsaturated acids and preferred the saturated acids. Surprisingly, FakB4 functioned with each of the fatty acids tested except octanoate. Hence, it seems that increased transcription/translation of only FakB4 (Figure S5) would allow incorporation of a wide palate of exogenous long‐chain fatty acids. A similar situation is seen in *S. pneumoniae* , where FakB3 functions with saturated and both monosaturated and diunsaturated acids (Gullett et al. 2019). Our findings are in marked contrast to the straightforward case in *S. aureus* , which has FakB1, a protein highly specific for saturated fatty acids, and a second, FakB2, selective for unsaturated acids (Cuypers et al. 2019).

A reasonable hypothesis is that subsets of the four *fakB* genes are expressed in different environments (e.g., the gut, biofilms, infection sites, fecal contaminated water). However, testing these environments would be difficult because they lack defined fatty acid compositions, and competing bacteria may be present. In arduous experiments, Rock and coworkers tested the role of *S. aureus* fatty acid uptake in a mouse wound model (Frank et al. 2020). They report that a lack of fatty acid activation had no effect on the growth of *S. aureus* in the wounds (both Δ*fakA* and Δ*fakB1* Δ*fakB2* strains were assayed).

The increased fatty acid synthesis resulting from a recessive mutation in *fabT* acts to suppress loss of FakA (Figure 4). The *fabT* mutation seems a straightforward loss of function mutation due to switching the charge of residue 44 from acidic to basic (glutamate to lysine) within a conserved sequence. Consistent with loss of function, the E44K mutation is recessive to the wild type allele in growth assays (Figure 8A) and the mutation results in the increased fatty acid synthesis seen in *∆fabT* strains (Zhu et al. 2019; Zou et al. 2022). This would bypass loss of the FakA fatty acid kinase because the increased acyl‐ACP synthesis would push the equimolar equilibrium of the PlsX acyl‐ACP: phosphate acyltransferase towards acyl‐phosphate. This would provide the acyl‐phosphate required by PlsY to catalyze the first acylation of the phospholipid synthesis pathway.

While this paper was under review, a contribution from the Fozo group (Johnston et al. 2025) appeared which reported the deletion of all four *E. faecalis* *fakB* genes plus strains having three or four *fakB* gene deletions. No attempts to delete *fakA* were reported. In agreement with our results, the Fozo lab reported that all ∆*fakB* deletion strains were viable, as was the strain lacking all four *fakB* genes. Moreover, their *∆fakBquad* strain was defective in the incorporation of exogenous oleate or palmitate into membrane lipids (Johnston et al. 2025) confirming our conclusion that these four proteins are the only FakBs. Fozo and coworkers reported cell morphology and growth rate phenotypes among the deletion strains when grown in BHI medium, but these largely disappeared when the strains were grown in a chemically defined medium. These differences may be due to the saturated fatty acids present in BHI medium (Johnston et al. 2025). Fozo and coworkers focused on phenotypic and bioinformatic aspects and did not address *fakB* regulation or the properties of purified FakB proteins (Johnston et al. 2025). Their data showed that all single and triple *∆fakB* strains were inhibited by a fatty acid addition to BHI medium, whereas their *∆fakBquad* strain showed no inhibition. Where the data overlap, there are only minor differences between our results and those of Fozo and coworkers. However, Fozo and coworkers reported that construction of their *∆fakBquad* required that the *tesE* thioesterase encoding gene must first be deleted (Johnston et al. 2025). However, our *∆fakBquad strain* has an intact *tesE* gene. This inconsistency may be due to strain differences between the Fozo OG1RF strain and our FA2‐2 strain, plus different growth media (Johnston et al. 2025). For example, although the two *E. faecalis* strains have very similar genome sizes, strain FA2‐2 fails to grow on the chemically defined medium used by Fozo and coworkers, perhaps because it lacks the 28 kb plasmid of OG1RF. A difficulty in *E. faecalis* research is the lack of a chemically defined medium that supports the growth of all strains of the bacterium.

Note that the *fakB5* of Fozo and coworkers (Johnston et al. 2025) and Waters and Eijkelkamp (Waters and Eijkelkamp 2024) is our *fakB3*. These workers skipped *fakB3* in *E. faecalis* because there was no homologue of *Streptococcus pneumoniae* *fakB3* which encodes a FakB strictly required for incorporation of linoleic acid, although its ability to accept other acids was not tested (Gullett et al. 2019). However, there seems to be no need for a homologue of *S. pneumoniae* FakB3 in *E. faecalis* because the strains containing only FakB2 or FakB1 showed good incorporation of [1‐^14^C]linoleic acid (Figure 6A). Moreover, in vitro, all purified FakB proteins except FakB3 showed good utilization of linoleic acid for acyl‐AcpB synthesis (Figure 7). It may be that consigning the *Streptococcus* and *E. faecalis* FakBs into tidy groupings could elicit less rather than greater clarity. This is especially so because the low sequence identities (27%–32%) characteristic of FakBs mean that clade placements are based on rather few residue differences which could give false inferences that clade denotes fatty acid selectivity. For example, Waters and Eijkelkamp (Waters and Eijkelkamp 2024) denote the FakB1 clade as specific for saturated fatty acids. However, the *E. faecalis* sequence placed in the FakB1 clade (also our FakB1) was almost nonfunctional with saturated fatty acids in vitro (Figure 7A) and the *∆fakB234* strain showed only modestly decreased [1‐^14^C] saturated fatty acid uptake (Figure 6B,C). Hence, although it is tempting to assign FakB specificity in one bacterial species to a similar protein in another species, this may not be warranted.

In *E. faecalis* , FakBs may play a role in endogenous fatty acid recycling in addition to the utilization of exogenous acids (Figure 8). A role in endogenous fatty acid recycling has been ascribed to *S. pyogenes* FabB4 (Lambert et al. 2023). A strain lacking *fabB4* was reported to incorporate exogenous fatty acids normally; hence, FabB4 was ascribed a recycling role. However, the *fabB4* strain contained three other FakBs which could readily account for the normal fatty acid uptake reported. Transcription of *fabB4* was reported to be regulated by FabT (Lambert et al. 2023). This result seems perverse because fatty acids would be converted to acyl‐ACPs, which would trigger FabT repression of *fakB4* transcription. Hence, fatty acids would block fatty acid utilization.

## 4 Materials and Methods

### 4.1 Materials

Fatty acids, antibiotics, and anhydrotetracycline (ahTC) were purchased from Sigma‐Aldrich. The media were purchased from Fisher Scientific. The DNA polymerase, restriction endonuclease, T4 ligase, and Gibson Assembly Cloning Kit were purchased from New England Biolabs. Sodium [1‐^14^C]acetate (specific activity, 57.0 mCi/mmol) was provided by Moravek Inc. [1‐^14^C]octanoate acid (specific activity, 57.0 mCi/mmol), [1‐^14^C]palmitic acid (specific activity, 55.0 mCi/mmol), [1‐^14^C]stearic acid (specific activity, 53.0 mCi/mmol) and [1‐^14^C]linoleic acid (specific activity, 55.0 mCi/mmol) were purchased from American Radiolabeled Chemicals. Ni‐NTA resin was from Invitrogen, and the DNA purification kits were from Qiagen. Silver nitrate silica gel thin layer plates were purchased from Analtech, and M17 Broth was from Becton Dickenson. Newborn calf serum was purchased from Thermo Fisher Scientific. All the other reagents were of the highest available quality. Oligonucleotide primers were synthesized by Integrated DNA Technologies, and DNA sequencing was performed by ACGT Inc.

### 4.2 Bacterial Strains, Plasmids, and Media

The bacterial strains and plasmids used in this study are listed in Table S2 and the primers used for this study are listed in Table S3. *E. coli* cultures were incubated at 37°C in Luria‐Bertani medium (tryptone, 10 g/L; yeast extract, 5 g/L; NaCl, 10 g/L) whereas *E. faecalis* cultures were grown at 37°C in M17 medium (BD Difco) or AC medium (tryptone, 10 g/L; yeast extract, 10 g/L; KH_2_PO_4_, 5 g/L; glucose, 1 g/L). Antibiotics were added at the following final concentrations (in μg/mL) for *E. coli* : kanamycin sulfate 30, erythromycin 250, chloramphenicol 30, and for *E. faecalis* : erythromycin 10; for *E. faecalis* CRISPr/cas12a: erythromycin 50 and anhydrotetracycline (ahTC) 0.25.

### 4.3 Construction of *E. faecalis fakA* and *fakB* Deletion Strains

The *E. faecalis fakB* single mutant strains were constructed via two sequential homologous recombination events using the methods described previously (Dong and Cronan 2022). Upstream and downstream ~500 bp DNA fragments were assembled by overlap PCR to generate the deletion cassette. For upstream of *fakB*, the primer *fakB1* up1 and *fakB1* dn1 were used for *fakB1*, the primer *fakB2* up1 and *fakB2* dn1 were used for *fakB2*, the primer *fakB3* up1 and *fakB3* dn1 were used for *fakB3*, and the primer *fakB4* up1 and *fakB4* dn1 were used for *fakB4*. For downstream of *fakB*, the primer *fakB1* up2 and *fakB1* dn2 were used for *fakB1*, the primer *fakB2* up2 and *fakB2* dn2 were used for *fakB2*, the primer *fakB3* up2 and *fakB3* dn2 were used for *fakB3*, and the primer *fakB4* up4 and *fakB4* dn4 were used for *fakB4*. The primer pBVGh up and pBVGh dn were used for linearization of the temperature‐sensitive vector pBVGh. The *fakB* deletion cassettes were inserted into pBVGh using the Gibson assembly kit (New England Biolabs). The resulting plasmids were transformed into competent cells of *E. faecalis* FA2‐2 by electroporation. One blue transformant colony was streaked on AC agar medium containing 10 μg/mL erythromycin and 0.1 mg/mL 5‐bromo‐4‐chloro‐3‐indolyl‐β‐D‐galactopyranoside (X‐Gal) and incubated overnight at 42°C to confirm the blue colony indicating chromosomal integration of the plasmid. One such single crossover strain was cultured in AC liquid medium at 30°C overnight. The final culture was diluted and plated on AC agar containing X‐Gal (0.1 mg/mL) and incubated for 24–48 h at 42°C. Colony PCR was used for analyzing the white colonies which represent double‐crossover events to give the deletion strain.

The *E. faecalis ∆fakA, ∆fakB* triple mutant strains and ∆*fakB* quad mutant were constructed using the CRISPr/cas12a system (Chua and Collins 2022). For the ∆*fakA* deletion, the upstream and downstream ~500 bp DNA fragments were assembled by overlap PCR to generate the deletion cassette. The primers *fakA* up1 and *fakA* dn1 were used for upstream, and the primers *fakA* up2 and *fakA* dn2 were used for downstream. For ∆*fakB*, the deletion cassettes were used as templates; the primers *fakB1* up and *fakB1* dn were used for *fakB1*, the primers *fakB2* up and *fakB2* dn were used for *fakB2*, the primers *fakB3* up and *fakB3* dn were used for *fakB3*, and the primers *fakB4* up and *fakB4* dn were used for *fakB4*. The primers pUC19 up and pUC19 dn were used for the linearization of the pUC19.pcRNP plasmid (containing the small RNA promoter and repeat region) for insertion of *fakA* and *fakB* deletion cassettes by the Gibson assembly kit to generate pUC19.pcRNP‐A and pUC19.pcRNP‐B. The CRISPR protospacer, which is a 23 bp spacer sequence homologous to the target sequence with a protospacer adjacent motif (PAM) of 5′‐TTTV‐3′ immediately upstream, was then inserted into pUC9.pcrRNP‐A or pUC9.pcrRNP‐B by overlap PCR. The primers *fakA* PAM up and *fakA* PAM dn were used for the insertion of *fakA* PAM using vector pUC9.pcrRNP‐A as the template; the primers fakB1 PAM up and fakB1 PAM dn were used for the insertion of *fakB1* PAM using vector pUC9.pcrRNP‐B1 as the template; the primers fakB2 PAM up and fakB2 PAM dn were used for the insertion of *fakB2* PAM using vector pUC9.pcrRNP‐B2 as the template; the primers fakB3 PAM up and fakB3 PAM dn were used for the insertion of *fakB3* PAM using vector pUC9.pcrRNP‐B3 as the template; the primers fakB4 PAM up and fakB4 PAM dn were used for the insertion of *fakB4* PAM using vector pUC9.pcrRNP‐B4 as the template. Then, the universal primers pUC19U up and pUC19U dn were used to amplify the PAM and deletion cassettes, which were inserted into the linearization vector pJC005 by primers pJC005 up and pJC005 dn using the Gibson assembly kit to generate pJC005‐A and pJC005‐B.

The plasmids (pJC005‐A and pJC005‐B) were transformed into competent cells of *E. faecalis* FA2‐2 by electroporation. The transformants were streaked on AC agar medium containing 50 μg/mL erythromycin and then cultured in AC liquid medium at 37°C overnight with 50 μg/mL erythromycin and 250 ng/mL ahTC (for induction of Cas12a expression). The final culture was diluted and plated on AC agar containing 50 μg/mL erythromycin and incubated for 24–48 h at 37°C. Colony PCR was used to screen the colonies for deletion strains. The deletion strains were cultured overnight in AC medium containing 250 ng/mL ahTC at 37°C. If required, this step was repeated three times to cure the Cas12a plasmid, and the final culture was diluted and plated on AC agar, incubated for 24–48 h at 37°C, screening for erythromycin sensitive colonies. For *fakA* mutant and *fakB* quad mutant screening, 20% bovine calf serum was added to the AC medium.

The *fakB* expression plasmids were constructed by inserting the *fakB* genes into a shuttle plasmid vector either pBM02(*aguR*) or pTRK L2(*aguR*) with an agmatine induced promoter through Gibson assembly. The plasmid pBM02(*aguR*) was linearized by primers pBM02 up and *aguR* dn. The plasmid pTRK L2(*aguR*) was linearized by primers pTRK L2(*aguR*) up and *aguR* dn. *E. faecalis* genome DNA as template, the *fakB* genes were amplified by the primers *fakB aguR* up and *fakB aguR* dn for fusing with pBM02(*aguR*) and the primers *fakB aguR* up and *fakB aguR* L2 dn for fusing with pTRK L2(*aguR*). Transformation of the *E. faecalis ∆fakBquad* mutant strain with these plasmids produced the complementation strains. *E. faecalis fabT* gene was amplified by primers *fabT aguR* up and *fabT aguR* dn, then fused with pBM02(*aguR*) through Gibson assembly. Transformation of the suppressor of *E. faecalis fakA* mutant strain with this plasmid produced the complementation strains.

### 4.4 Expression and Purification of His_6_ Tagged FakA and FakB Proteins

The expression vector pET28(b) carrying either *fakA* or a *fakB* was constructed as given previously (Zou et al. 2022). The expression vectors carrying *fakA* and *fakB1* were transformed into strain BL21(DE3) and the others were transformed into the *E. coli* Rosetta strain. The transformants were incubated in medium at 37°C with 30 μg/mL kanamycin to an OD_600_ of 0.6 and then were induced by 1 mM IPTG for another 4 h incubation. The cells were harvested and lysed in lysis buffer (50 mM sodium phosphate (pH 7.0) 300 mM NaCl and 10 mM imidazole). The supernatants were loaded onto the Ni‐NTA column. The columns were eluted with a wash buffer (50 mM sodium phosphate (pH 7.0) 300 mM NaCl and 40 mM imidazole) and the tagged proteins were then eluted with the same buffer containing 250 mM imidazole. The eluted proteins were dialyzed against 50 mM sodium phosphate (pH 7.0) and 300 mM NaCl. Glycerol was added after dialysis to 15%, and the proteins were stored at −80°C.

### 4.5 FakB Selectivity Assays

FakB ligand binding assays followed the protocol of (Parsons et al. 2014). Substrate selectivity of FakB1, FakB2, FakB3, and FakB4 was examined by incubating FakB with different radiolabeled fatty acids. The reactions (200 μL) consisted of 6.3 μM of FakB and 1.25 μM [1‐^14^C]‐labeled fatty acid in PBS, pH 7.4. Reactions were incubated at room temperature for 10 min before the addition of 200 μL PBS and removal of unbound fatty acid using a 2 mL Zeba spin desalting column (Thermo Scientific). The elution fractions were analyzed by scintillation counting.

### 4.6 Acyl‐AcpB Final Product Assays

*E. faecalis* AcpB was converted into functional holo‐AcpB using the methods described previously (Zhu et al. 2019). To synthesize *E. faecalis* acyl‐ACP species, holo‐AcpB was mixed with various 50 μM free fatty acids irrespectively in the presence of *E. faecalis* 2 μM FakA, 0.17 μM FakB, and 2 μM PlsX proteins in buffer containing 50 mM Tris–HCl (pH 7.5), 2 mM MgCl_2_, 1 mM DTT, and 0.2 mM ATP. The reaction was initiated by adding FakB and was incubated at room temperature for 2 min or 4 min. The reactions were stopped by adding an equal volume of 10 M urea and placed on dry ice (Cao et al. 2017). The products were analyzed on 18% PAGE gels containing 2.5 M urea.

### 4.7 Growth Measurements of *E. faecalis* Strains

The growth curves of *E. faecalis* wild type, *fakA* mutant, and *fakB* mutant strains were tested in M17 medium at 37°C. The OD_600_ values of the culture were measured every 1.5 h in triplicate for 10.5 h. For the growth phenotypes of the *fakA* mutant and *fakB* mutant strains with different chain fatty acids, *E. faecalis* wild type, *fakA* mutant, and *fakB* mutant were streaked on M17 agarose medium containing different chain length fatty acids for 2 days.

### 4.8 Thin‐Layer Chromatography Analysis of Phospholipid Acyl Chains and Fatty Acids in Culture Media

Note that although *E. faecalis* contains two glucosyl lipids (diglucosyl‐diacylglycerol and monoglucosyl‐diacylglycerol), in these analyses we have considered these as phospholipids because the acyl chains of these lipids are incorporated as phosphatidic acid. For assays of *de novo* fatty acid biosynthesis in the presence of exogenous oleate, *E. faecalis* wild type and *fakB* mutant were started at an optical density (OD) of 0.3 in M17 medium, followed by incubation at 37°C in the presence of 1 μCi/mL [1‐^14^C]acetate for 3 h with 0.1 mM oleate. After labeling, the cells were collected and lysed with methanol‐chloroform (2:1) solution, and the phospholipids were extracted in chloroform and dried under nitrogen. The fatty acyl groups were methylated by transesterification in 25% (wt/vol) sodium methoxide, extracted by hexanes, and processed for thin‐layer chromatography (TLC) analysis on Analtech silica gel G plates containing 20% silver nitrate in toluene at −20°C. The plates containing the ^14^C‐labeled fatty acid methyl esters were exposed for phosphorimager analysis on a GE Typhoon FLA700 Scanner, and the data were analyzed using ImageQuant TL software.

To test exogenous fatty acid incorporation, *E. faecalis* wild type, *fakA* mutant, *fakB* mutant, and complementary strains were inoculated at an OD of 0.3 in M17 medium, followed by incubation at 37°C in the presence of 0.1 μCi/mL [1‐^14^C] fatty acid (for octanoic acid 1 mCi/L) for 3 h (octanoic acid 6 h). After labeling, the cells were collected and washed twice with sterile H_2_O, then normalized to a final concentration. The normalized final cell suspensions were lysed with methanol‐chloroform (2:1) solution, and the phospholipids were extracted in chloroform and then dried under nitrogen. The fatty acyl groups were methylated by 25% (wt/vol) sodium methoxide, extracted by hexanes, and processed for thin‐layer chromatography (TLC) analysis on Analtech silica gel containing 20% silver nitrate developed in toluene at −20°C. The plates containing the ^14^C‐labeled fatty acid methyl esters were exposed for phosphorimaging analysis using a GE Typhoon FLA700 Scanner, and the data were analyzed using ImageQuant TL software.

To test the possibility of fatty acid recycling, the *E. faecalis* wild type, *∆fakA*, ∆*fakA* supp, *∆fakBquad*, and *∆fakBquad* supp strains were inoculated into AC medium (initial OD 0.15), followed by incubation at 37°C in the presence of 1 μCi/mL [1‐^14^C]acetate for 30 h. After labeling, glacial acetic acid (0.15 mL) was added to break down any fatty acid salts, followed by 9 mL of methanol‐chloroform (2:1) to extract cells and medium together. The lipids were dried under nitrogen, spotted on Analtech silica gel G plates, and developed twice as described in Figure 7. The plates containing the ^14^C‐labeled lipids were exposed for phosphorimaging analysis using a GE Typhoon FLA700 Scanner, and the data were analyzed using ImageQuant TL software.

### 4.9 Gas Chromatography–Mass Spectrometry Analysis of the Fatty Acids of Cell Membrane Phospholipids

*E. faecalis fakA* mutant and *fakB* mutant strains were inoculated at logarithmic phase in M17 medium with or without 0.1 mM oleic acid. The cells were lysed by methanol‐chloroform (2:1) solution, and the phospholipids were extracted into chloroform and dried under a nitrogen stream. The fatty acyl groups were methylated by transesterification using 25% (wt/vol) sodium methoxide in methanol, extracted with hexanes, and submitted to the Carver Metabolomics Center of the University of Illinois at Urbana‐Champaign for gas chromatography–mass spectrometry analysis.

### 4.10 Extraction of Total RNA, cDNA Synthesis and Real‐Time Reverse Transcription‐Quantitative PCR(RT‐qPCR)

Total RNA of *E. faecalis* was purified using RNeasy Mini Kit (Qiagen). RNAs were non‐specifically converted to single‐stranded cDNAs using the ProtoScript First Strand cDNA Synthesis Kit (NEB Biolabs). The resulting cDNA served as the template for qRT‐PCR. The RT‐qPCR assay was conducted using iQ SYBR green Supermix (Bio‐Rad) with the 16S RNA gene as an internal control using the primers RT *fakA*, RT *fakB*, and RT 16S (Table S3).

### 4.11 Translational Fusion Constructions

To measure the levels of translation of the *E. faecalis* *fakB* genes, the ribosome binding site together with the 5′ coding region for each *fakB* gene (−65 to +35 relative to the initiation codon ATG of each *fakB*) was first amplified by primer sets EffkaB1 RBS F and EffkaB1 ′ *SalI* R, EffkaB2 RBS F and EffkaB2 ′ *SalI* R, EffkaB3 RBS F and EffkaB3 ′ *SalI* R, and EffkaB4 RBS F and EffkaB4 ′ *SalI* R, respectively, and then ligated to the low activity *L. lactis* p32 promoter amplified by p32 PstI F and p32 R through overlap PCR. The constructed translation fusion segments for each *fakB* were then inserted at the 5′ end of the promoterless *lacZ* gene of plasmid pBHK322 (Bi et al. 2014) and transformed into *E. faecalis* wild‐type strain for assaying β‐galactosidase expression as in the previous work (Zou et al. 2022). The data were collected in triplicate. The fusion junction of each translational fusion was validated by sequencing.

## Author Contributions

**Huijuan Dong:** conceptualization, investigation, writing – original draft, methodology, writing – review and editing, validation. **Qi Zou:** investigation, writing – original draft, methodology, validation, writing – review and editing. **John E. Cronan:** conceptualization, investigation, funding acquisition, writing – original draft, writing – review and editing, validation, methodology, visualization.

## Ethics Statement

The authors have nothing to report.

## Conflicts of Interest

The authors declare no conflicts of interest.

## Supporting information


**Data S1:** mmi70017‐sup‐0001‐Supinfo.docx.

## Data Availability

The data that support the findings of this study are available from the corresponding author upon reasonable request.
