# Supplementary material for: Defining the Enterococcus faecalis Fatty Acid Kinase System of Exogeneous Fatty Acid Utilization
Source: Mol Microbiol. 2025 Aug 2;124(5):400–12. doi: 10.1111/mmi.70017 (PMC12594616; doi:10.1111/mmi.70017)
Supplement: Supplementary file 1 — Data S1: mmi70017‐sup‐0001‐Supinfo.docx. [file MMI-124-400-s001.docx]

Supplement to:

Defining the *Enterococcus faecalis* Fatty Acid Kinase System of Exogeneous Fatty Acid

Utilization

Huijuan Dong, Qi Zou and John E. Cronan


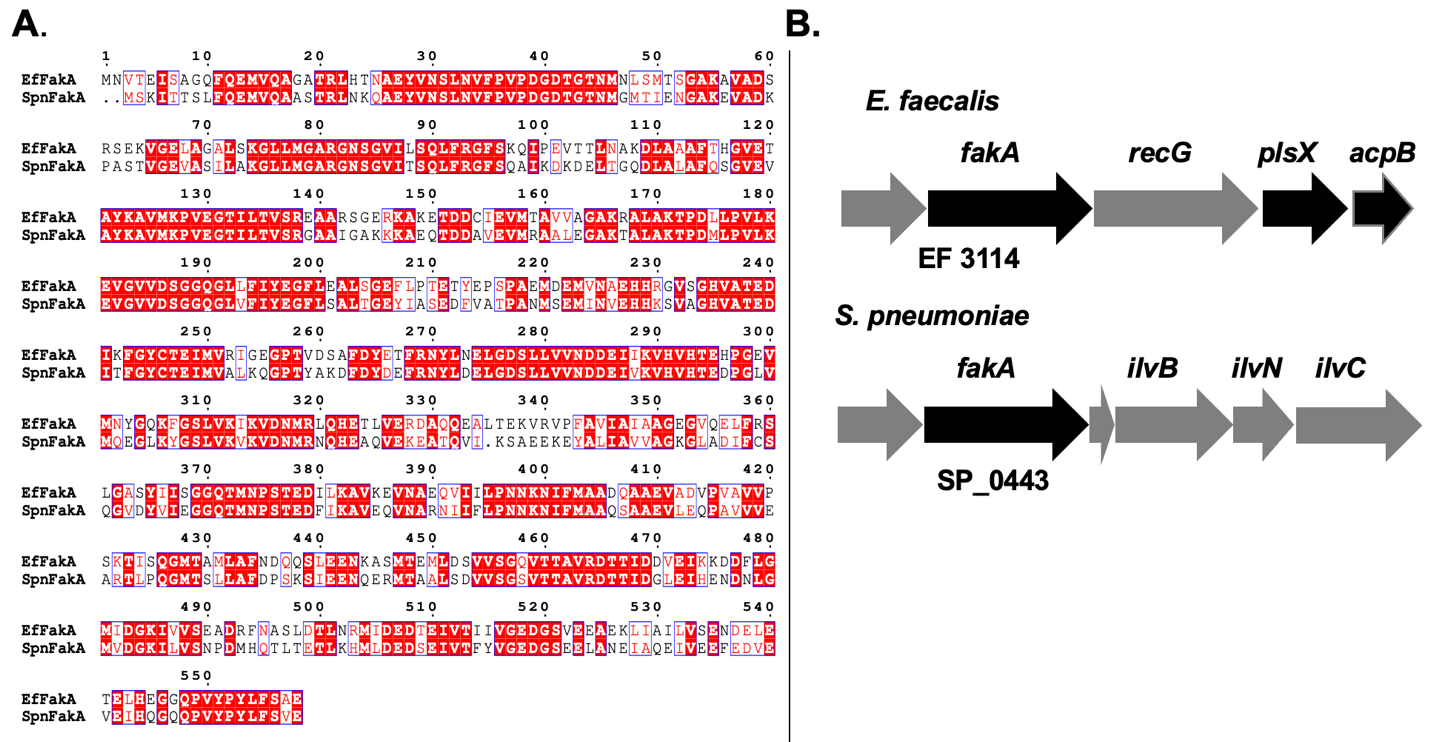


Fig. S1. The FakA proteins of *E. faecalis* and *S. pneumoniae*

***fakA***

***recG***

***plsX***

***acpB***

**EF 3114**

***E. faecalis***

***fakA***

***ilvB***

***ilvN***

***ilvC***

**SP_0443**

***S. pneumoniae***


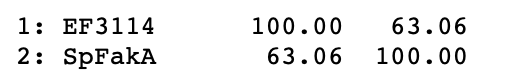


**A.** Alignments of the *E. faecalis* and *S. pneumoniae* FakA proteins. The two proteins are 63% identical. **B.** The differing genome neighborhoods of the two *fakA* genes.


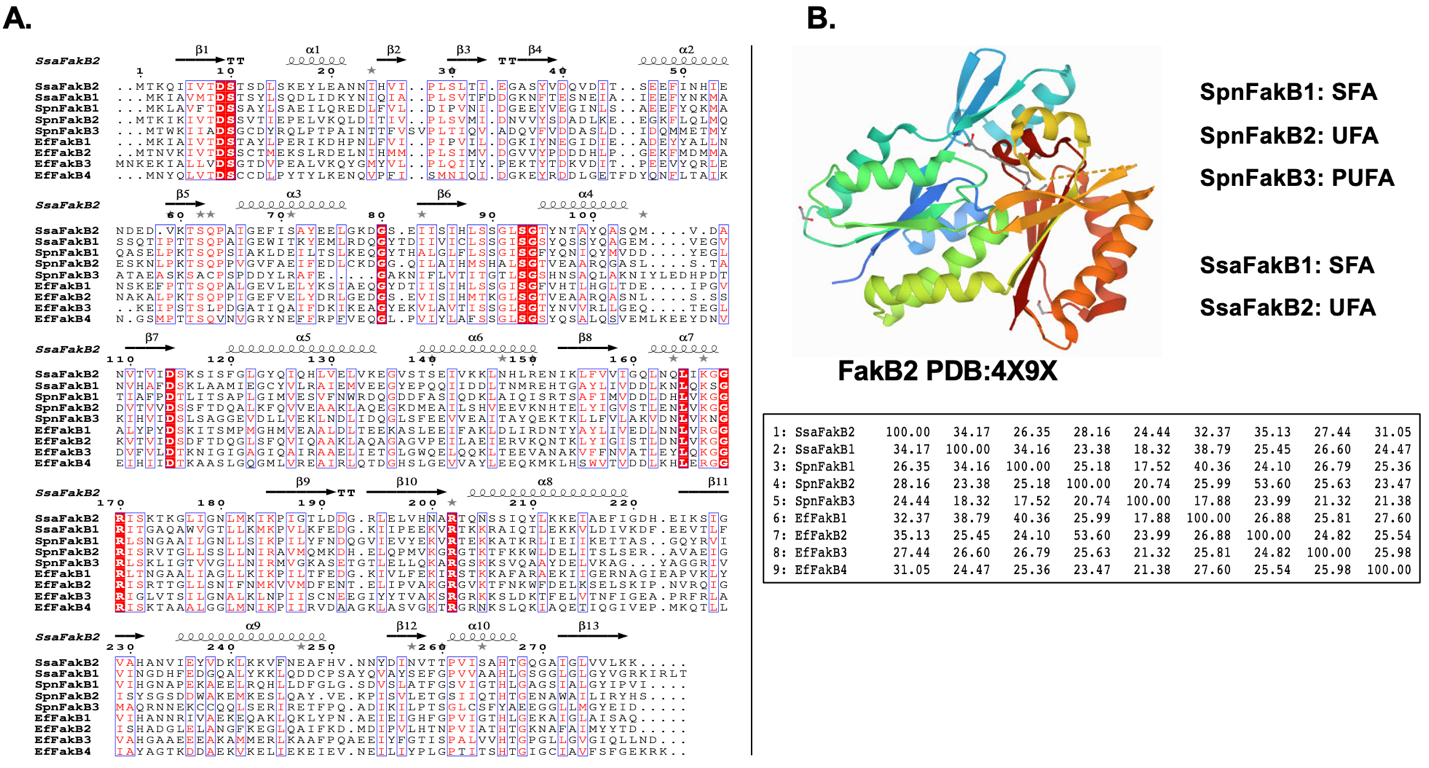


Fig S2. Alignment of FakB proteins

**A.** The proteins are those of the *Staphylococcus aureus* (Ssa), *S. pneumoniae* (Spn) and *E. faecalis* (Ef). The FakB protein family is Pfam02645. **B.** The 1.2-Å x-ray structure of *S. aureus* FakB2 and the identical residue values compared with all other FakBs. Each protein is given a value of 100 to allow comparison to all others.

Fig. S3. Deletion mutations constructed in the *E. faecalis* *fakA* and in all four *fakB* genes. All genes were deleted in frame except those the sequences remaining in ∆*fakB1* and ∆*fakB4*. These lack opening reading frames, but do not affect downstream gene expression.


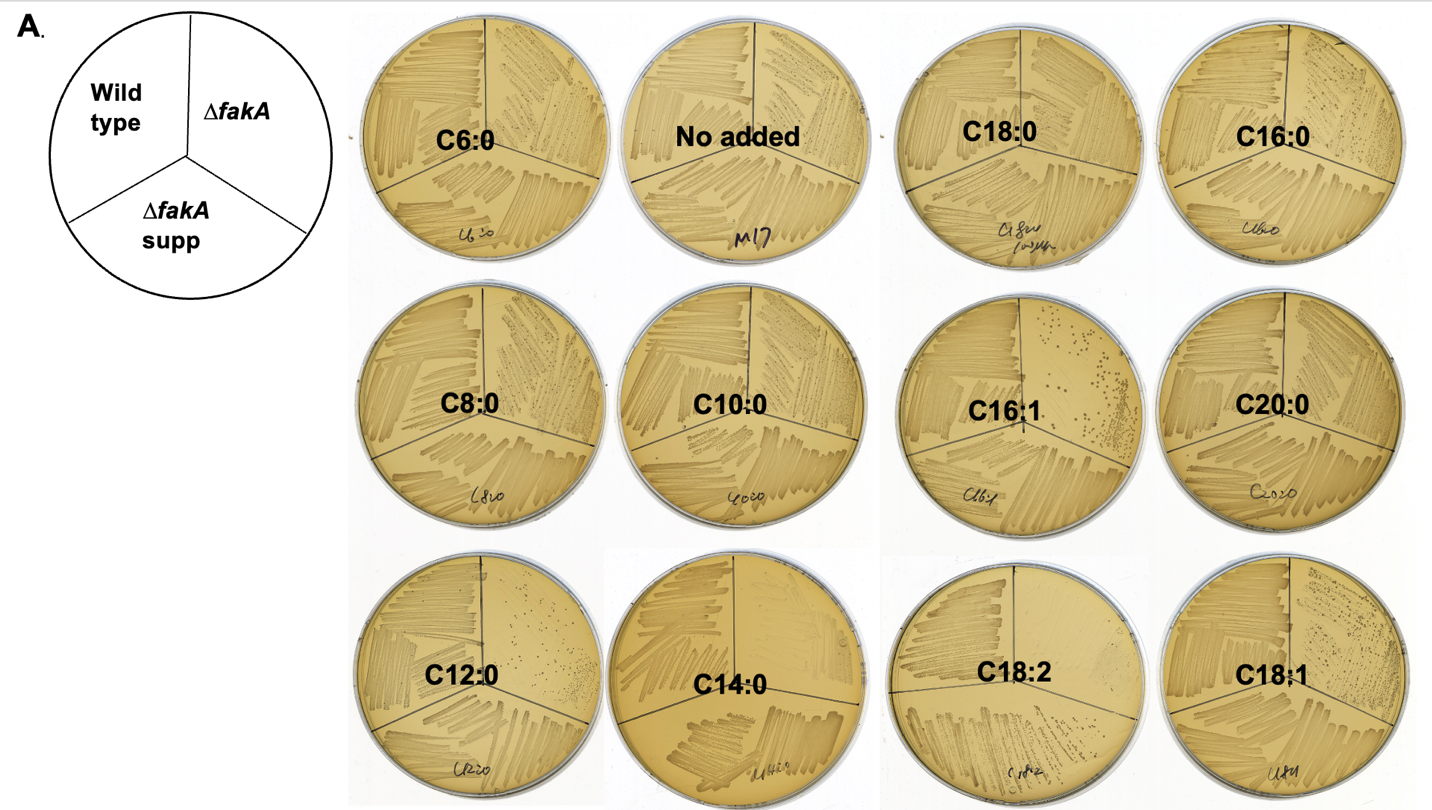


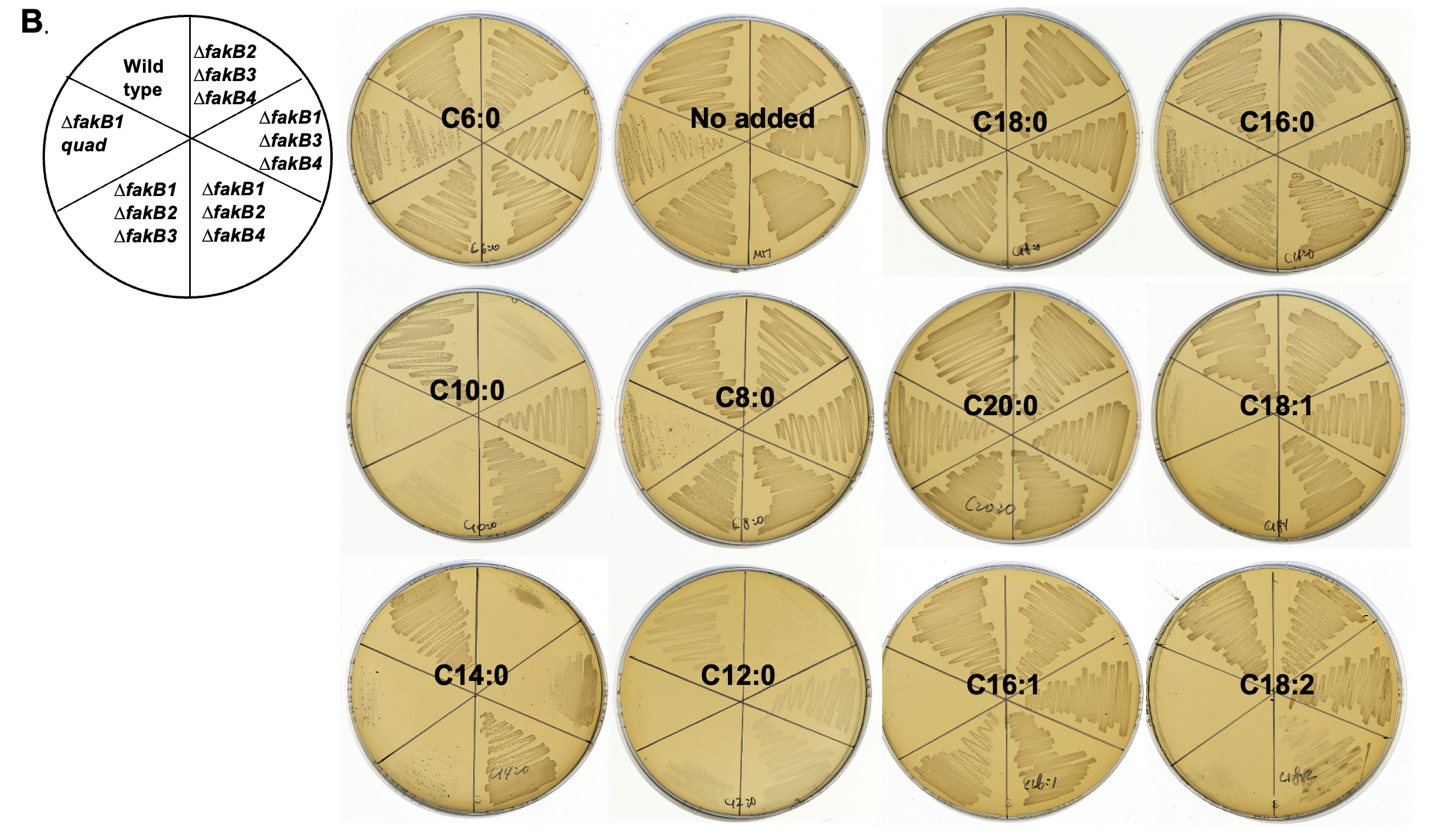


Fig. S4. Growth phenotypes of wild type, *∆fakA* and *∆fakB* strains on M17 plates with diverse fatty acids*.* **A.** Phenotypes of the wild type*, ∆fakA and ∆fakA* suppressor (sup) strains. **B.** Phenotypes of the wild type and *∆fakB* strains. Note: Hexanoic acid (C6:0), octanoic acid (C8:0), decanoic acid (C10:0), dodecanoic acid (C12:0), hexadecanoic acid (C16:0), octadecanoic acid (C18:0) oleic acid (C18:1) and eicosanoic acid (C20:0) were added at final concentrations of 0.1 mM in M17 agarose medium whereas tetradecanoic acid(C14:0) was added at 5 µM, palmitoleic acid (C16:1) was added at 10 µM and linoleic acid (C18:2) was added at 50 µM. The plates were incubated for 2-3 days at37°C depending on the fatty acid.


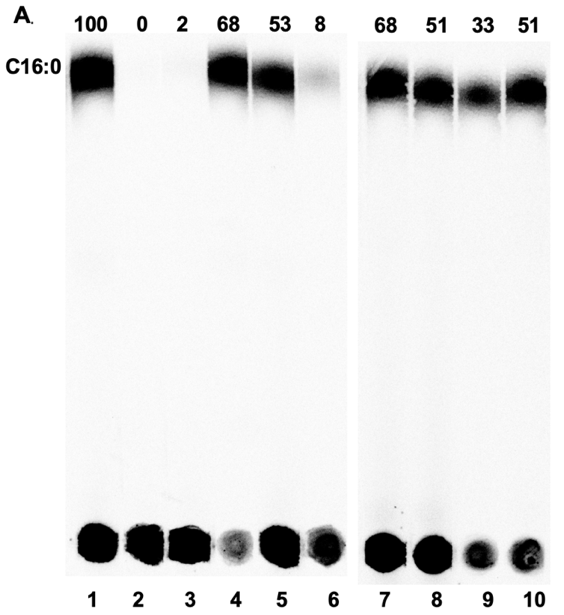

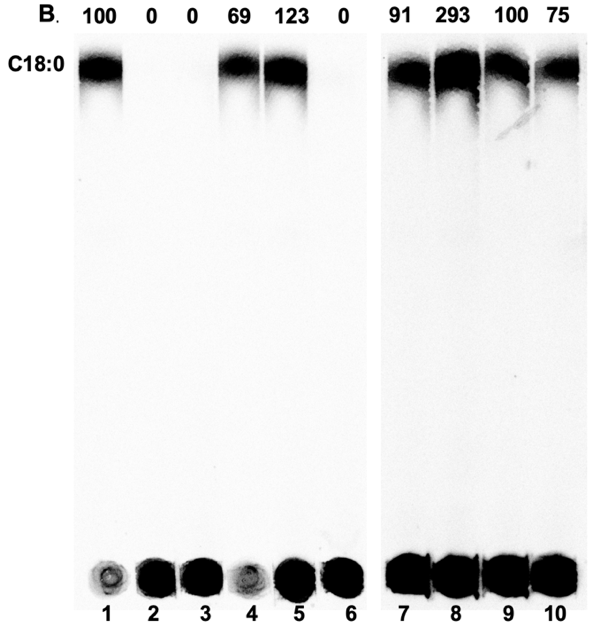


Fig. S5. Complementation of the *∆fakB quad* strain with wild type FakB genes.

**A**. The cultures were labeled with [1-^14^C]palmitic acid. **B**. The cultures were labeled with [1-^14^C]stearic acid. The numbers above the lanes given the radioactivity relative the that of the wild type strain (value of 100). The line between lanes 6 and 7 result because two plates were loaded and run in parallel. The complementation plasmids were either low copy (lc) or high copy (hc) with expression driven by the agmatine induced promoter. Expression from the high copy plasmid is approximately 80-fold greater than that given by the low copy plasmid based on *E. coli* β-galactosidase expression.

The lanes are:

1. wild type

2. *∆fakBquad*

3. *∆fakBquad* complemented with *fakB1* (lc)

4. *∆fakBquad* complemented with *fakB2* (lc)

5. *∆fakBquad* complemented with *fakB3* (lc)

6. *∆fakBquad* complemented with *fakB4* (lc)

7. *∆fakBquad* complemented with *fakB1* (hc)

8. *∆fakBquad* complemented with *fakB2* (hc)

9. *∆fakBquad* complemented with *fakB3* (hc)

10. *∆fakBquad* complemented with *fakB4* (hc)


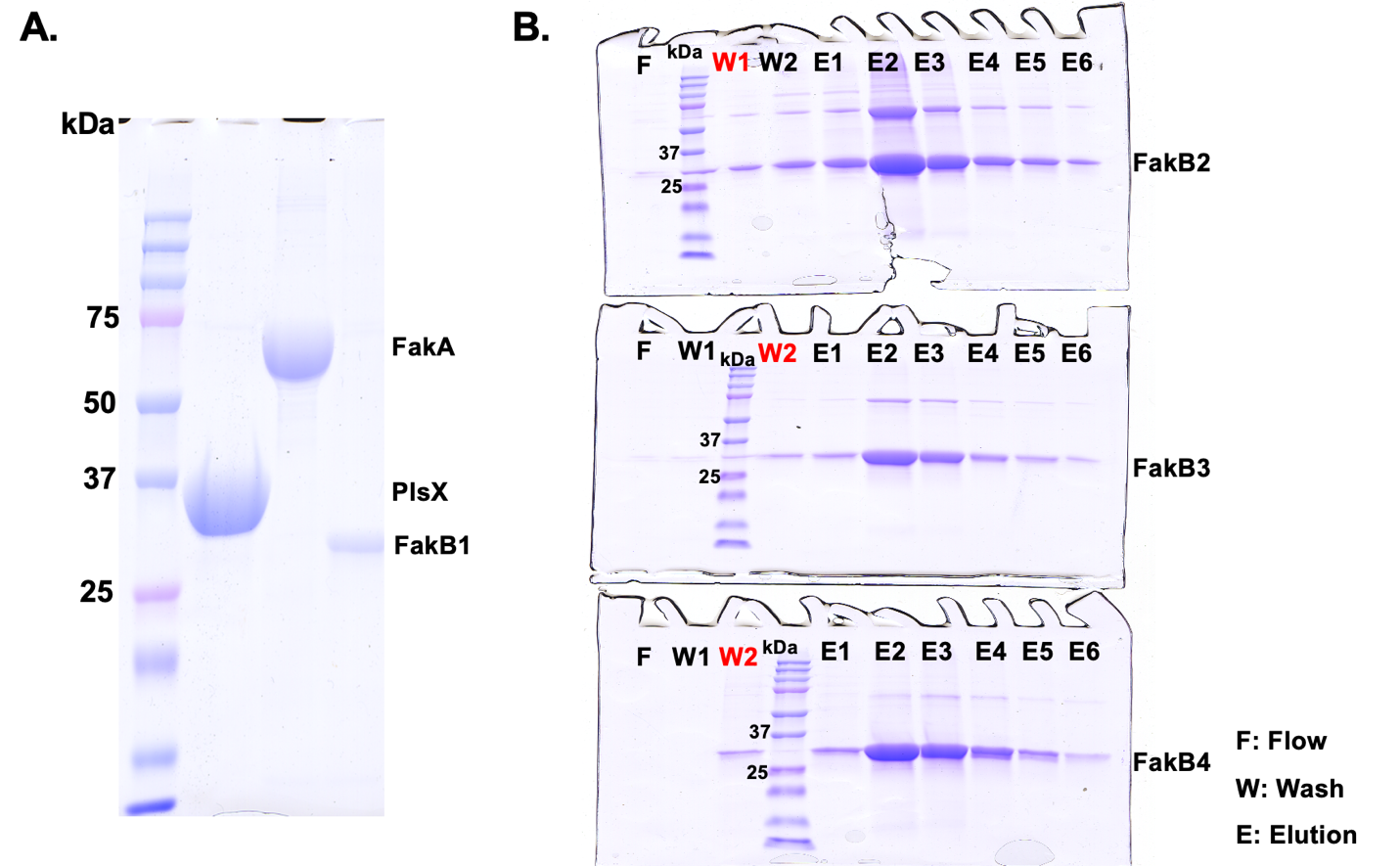


Fig. S6. Purification of the proteins used in the *in vitro* acyl-AcpB synthesis assay. A. SDS-PAGE gel analysis of purified samples of His-tagged PlsX, FakA and FakB1. The protein masses are consistent with the calculated values. B. Elution profiles of the FakB2- 4 proteins from the Ni chelate column. F: flowthrough fraction, W1-2: wash fractions, E: elution fraction, and W1 of FakB2 and W2 of FakB3 and FakB4 were used for the assay.

Table S1. Binding of [1-^14^C] fatty acids by purified FakB proteins. The assay was as described previously with the *Staphylococcus aureus* FakBs (Parsons *et al.*, 2014). The values are counts per min eluted from the separation column. The [1-^14^C] fatty acids have essentially the same specific activities (Materials and Methods) so these data can be directly compared.

| **Fatty acid** | **FakB1** | **FakB2** | **FakB3** | **FakB4** |
| --- | --- | --- | --- | --- |
| **Blank** | **17.17** | **18.83** | **13.83** | **14.83** |
| **[1-^14^C]C6:0** | **59.50** | **43.17** | **21.67** | **165.33** |
| **[1-^14^C]C8:0** | **46.67** | **27.17** | **17.17** | **74.33** |
| **[1-^14^C]C16:0** | **735.50** | **20.33** | **50.00** | **702.00** |
| **[1-^14^C]C18:0** | **1190.17** | **590.33** | **2319.00** | **541.33** |
| **[1-^14^C]C18:1(∆9)** | **559.67** | **1337.83** | **3016.17** | **249.50** |
| **[1-^14^C]C18:2(∆9,∆12)** | **64.50** | **244.17** | **1654.50** | **163.83** |

**Table S2. Strains and Plasmids**

| **Strains or Plasmids** | **Relevant characteristics** | | **Reference or source** | |  |
| --- | --- | --- | --- | --- | --- |
| ***E. coli* strains** |  | |  | |  |
| DH5α | φ80d lacZΔM15 endA1recA1hsdR17(rK-, mK+) | | Lab stock | |  |
| EC1000 | Em^R^; MC1000 derivative carrying a single copy of the pWV01 *repA* gene in *glgB* | | Lab stock | |  |
| BL21(DE3) | *omp*T *hsd*S B (rB^-^ mB^-^) | | Lab stock | |  |
| Rosetta (DE3) | *ompT hsdSB* (rB^-^ mB^-^) *gal dcm* (DE3) pRARE (Cm^r^) | | Lab stock | |  |
| ***E. faecalis* strains** |  | |  | |  |
| FA2-2 | Wild type | | Lab stock | |  |
| HJ573 | *∆fakB1* | | This work | |  |
| HJ577 | *∆fakB2* | | This work | |  |
| HJ666 | *∆fakB3* | | This work | |  |
| HJ667 | *∆fakB4* | | This work | |  |
| HJ5 | *∆fakB2∆fakB3∆fakB4* | | This work | |  |
| HJ70 | *∆fakB1∆fakB3∆fakB4* | | This work | |  |
| HJ18 | *∆fakB1∆fakB2∆fakB4* | | This work | |  |
| HJ65 | *∆fakB1∆fakB2∆fakB3* | | This work | |  |
| HJ6 | *∆fakB1∆fakB2∆fakB3∆fakB4 (fakBquad)* | | This work | |  |
| HJ93 | *∆fakA* | | This work | |  |
| HJ185 | *∆fakBquad* carrying pBM02 *aguRfakB1* | | This work | |  |
| HJ186 | *∆fakBquad* carrying pBM02 *aguRfakB2* | | This work | |  |
| HJ187 | *∆fakBquad* carrying pBM02 *aguRfakB3* | | This work | |  |
| HJ197 | *∆fakBquad* carrying pBM02 *aguRfakB4* | | This work | |  |
| HJ253 | *∆fakBquad* carrying pTRK L2 *aguRfakB1* | | This work | |  |
| HJ269 | *∆fakBquad* carrying pTRK L2 *aguRfakB2* | | This work | |  |
| HJ13 | *∆fakBquad* carrying pTRK L2 *aguRfakB3* | | This work | |  |
| HJ270 | *∆fakBquad* carrying pTRK L2 *aguRfakB4* | | This work | |  |
| QZ639 | *L. lactis* p32 promoter plus *E. faecalis fakB1* start region (-65 to +35) at 5'-end of *lacZ* in pBHK322 | | This work | |  |
| QZ640 | *L. lactis* p32 promoter plus *E. faecalis fakB2* start region (-65 to +35) at 5'-end of *lacZ* in pBHK322 | | This work | |  |
| QZ641 | *L. lactis* p32 promoter plus *E. faecalis fakB3* start region (-65 to +35) at 5'-end of *lacZ* in pBHK322 | | This work | |  |
| QZ642 | *L. lactis* p32 promoter plus *E. faecalis fakB4* start region (-65 to +35) at 5'-end of *lacZ* in pBHK322 | | This work | |  |
| **Plasmids** |  | |  | |  |
| pBVGh | Em^R^, shuttle vector carrying pWV01 replicon (Ts) and *E. coli lacZ* | | Lab stock | |  |
| pET28(b) | Kan^r^, expression plasmid | | Lab stock | |  |
| pBM02(*aguR*) | Em^R^, high-copy-no. shuttle vector with an *aguR* promoter | | Lab stock | |  |
| pTRK L2(*aguR*) | Em^R^, low-copy-no. shuttle vector with an *aguR* promoter | | Lab stock | |  |
| pUC.sRNAP | pUC, *bla*; small RNA promoter and 23-bp spacer sequence flanked by two 19-bp repeats | | (1) | |  |
| pJC005 | Em^R^, CRISPR-Cas12a genome editing plasmid | | (1) | |  |
| pBVGhB1 | Em^R^, *E. faecalis fakB1* deletion cassette in pBVGh | | This work | |  |
| pBVGhB2 | Em^R^, *E. faecalis fakB2* deletion cassette in pBVGh | | This work | |  |
| pBVGhB3 | Em^R^,  *E. faecalis fakB3* deletion cassette in pBVGh | | This work | |  |
| pBVGhB4 | Em^R^, *E. faecalis fakB4* deletion cassette in pBVGh | | This work | |  |
| pJC005B1 | Em^R^, *E. faecalis fakB1* deletion cassette and PAM in pJC005 | | This work | |  |
| pJC005B2 | Em^R^, *E. faecalis fakB2* deletion cassette and PAM in pJC005 | | This work | |  |
| pJC005B3 | Em^R^, *E. faecalis fakB3* deletion cassette and PAM in pJC005 | | This work | |  |
| pJC005B4 | Em^R^, *E. faecalis fakB4* deletion cassette and PAM in pJC005 | | This work | |  |
| pJC005A | Em^R^, *E. faecalis fakA* deletion cassette and PAM in pJC005 | | This work | |  |
| pBM02*aguR*B1 | Em^R^, *E. faecalis fakB1* in high copy vector pBM02(*aguR*) | | This work | |  |
| pBM02*aguR*B2 | Em^R^, *E. faecalis fakB2* in high copy vector pBM02(*aguR*) | | This work | |  |
| pBM02*aguR*B3 | Em^R^, *E. faecalis fakB3* in high copy vector pBM02(*aguR*) | | This work | |  |
| pBM02*aguR*B4 | Em^R^, *E. faecalis fakB4* in high copy vector pBM02(*aguR*) | | This work | |  |
| pTRK L2*aguR*B1 | Em^R^, *E. faecalis fakB1* in low copy vector pTRK L2(*aguR*) | | This work | |  |
| pTRK L2*aguR*B2 | Em^R^, *E. faecalis fakB2* in low copy vector pTRK L2(*aguR*) | | This work | |  |
| pTRK L2*aguR*B3 | Em^R^, *E. faecalis fakB3* in low copy vector pTRK L2(*aguR*) | | This work | |  |
| pTRK L2*aguR*B4 | Em^R^, *E. faecalis fakB4* in low copy vector pTRK L2(*aguR*) | | This work | |  |
| pBM02*aguRfabT* | Em^R^, *E. faecalis fabT* in high copy vector pBM02(*aguR*) | | This work | |  |
| pET28(b)-B1 | Kan^R^, *E. faecalis fakB1* in expression vector pET28(b) | | Lab stock | |  |
| pET28(b)-B2 | Kan^R^, *E. faecalis fakB2* in expression vector pET28(b) | | Lab stock | |  |
| pET28(b)-B3 | Kan^R^, *E. faecalis fakB3* in expression vector pET28(b) | | Lab stock | |  |
| pET28(b)-B4 | Kan^R^, *E. faecalis fakB4* in expression vector pET28(b) | | Lab stock | |  |
| pET28(b)-A | Kan^R^, *E. faecalis fakA* in expression vector pET28(b) | | This work | |  |
| pBHK322 | | promoterless *E. coli* *lacZ* on vector pTRKL2 | | Bi et al., 2014 | |
| pQZ635 | | Translational *fakB1-lacZ* fusion | | This work | |
| pQZ636 | | Translational *fakB2-lacZ* fusion | | This work | |
| pQZ637 | | Translational *fakB3-lacZ* fusion | | This work | |
| pQZ638 | | Translational *fakB4-lacZ* fusion | | This work | |

**Table S3. Oligonucleotide primers**

| primers* | Sequence 5’-3’ |
| --- | --- |
| pBVGh up | CTGCAGCCCGGGGGAT |
| pBVGh dn | GAGCTCCCGGGTACCATG |
| Ef *fakB1* up1 | ATCGATGCATGCCATGGTACCCGGGAGCTCAGGTGTCAAAATCGATGCAGA |
| Ef *fakB1* dn1 | GACTGGGATGGGAATTACAA |
| Ef *fakB1* up2 | ACTTTTTTAGAAAGTTTGAAAACAGGGAATATGCTTTAAGTAACTCGCTAG |
| Ef *fakB1* dn2 | TAGAACTAGTAGGGATCCCCCGGGCTGCAGATGTTTTGCAGCCGGAATTAC |
| Ef *fakB2* up1 | ATCGATGCATGCCATGGTACCCGGGAGCTCGATGGCGATTAACCAGACTA |
| Ef *fakB2* dn1 | AACGCCATCAACCATAATTGA |
| Ef *fakB2* up2 | CAATTATGGTTGATGGCGTTGACATGGATATTCCCGTATTACA |
| Ef *fakB2* dn2 | TAGAACTAGTAGGGATCCCCCGGGCTGCAGTTGATCACTACGATCCCCAT |
| Ef *fakB3* up1 | ATCGATGCATGCCATGGTACCCGGGAGCTCCAACAATTGGAAAACGTTCA |
| Ef *fakB3* dn1 | TTGTTTGACTAATGCCTCTG |
| Ef *fakB3* up2 | CAGAGGCATTAGTCAAACAATTTGGAACAATTAGTCCGGC |
| Ef *fakB3* dn2 | TAGAACTAGTAGGGATCCCCCGGGCTGCAGCAAATGAGGCAATCAAGCCA |
| Ef *fakB4* up1 | ATCGATGCATGCCATGGTACCCGGGAGCTCTTGATTCAGTAGTCATGCGTCA |
| Ef *fakB4* dn1 | ATCATCGCGATATTCTTTCCCG |
| Ef *fakB4* up2 | GGAAAGAATATCGCGATGATTATCCTCTAGGACCCACCAT |
| Ef *fakB4* dn2 | TAGAACTAGTAGGGATCCCCCGGGCTGCAGTGGTTACGTCAATTTGCTGGT |
| pUC19 up | CTCGAGGCCTGCAGACAT |
| pUC19 dn | ATCTACAAGAGTAGAAATTCATATCCATATGC |
| Ef *fakA* up1 | ATATGGATATGAATTTCTACTCTTGTAGATATGTGCTCTTATAAGGAGGCTTT |
| Ef *fakA* dn1 | ATGGATACACTCTAGACTGACCTGCGCTGATTT |
| Ef *fakA* up2 | CGCAGGTCAGTCTAGAGTGTATCCATACTTATTCTCAGCA |
| Ef *fakA* dn2 | TGCCAAGCTTGCATGTCTGCAGGCCTCGAGTGGCATCCCATTTGCC |
| Ef *fakB1* up | ATATGGATATGAATTTCTACTCTTGTAGATAGGTGTCAAAATCGATGCAGA |
| Ef *fakB1* dn | TGCCAAGCTTGCATGTCTGCAGGCCTCGAGATGTTTTGCAGCCGGAATTAC |
| Ef *fakB2* up | ATATGGATATGAATTTCTACTCTTGTAGATGATGGCGATTAACCAGACTA |
| Ef *fakB2* dn | TGCCAAGCTTGCATGTCTGCAGGCCTCGAGTTGATCACTACGATCCCCAT |
| Ef *fakB3* up | ATATGGATATGAATTTCTACTCTTGTAGATCAACAATTGGAAAACGTTCA |
| Ef *fakB3* dn | TGCCAAGCTTGCATGTCTGCAGGCCTCGAGCAAATGAGGCAATCAAGCCA |
| Ef *fakB4* up | ATATGGATATGAATTTCTACTCTTGTAGATTTGATTCAGTAGTCATGCGTCA |
| Ef *fakB4* dn | TGCCAAGCTTGCATGTCTGCAGGCCTCGAGTGGTTACGTCAATTTGCTGGT |
| Ef *fakA* PAM up | agattcgtgaagcggcgcgttctggtgAATTTCTACTCTTGTAGATatgtg |
| Ef *fakA* PAM dn | caccagaacgcgccgcttcacgaATCTACAAGAGTAGAAATTATGG |
| Ef *fakB1* PAM up | agatCAAATCAATCGCTGAACAAGGGTAATTTCTACTCTTGTAGAT |
| Ef *fakB1* PAM dn | ACCCTTGTTCAGCGATTGATTTGATCTACAAGAGTAGAAATTATGG |
| Ef *fakB2* PAM up | agatTAGAATTATATGATCGCTTAGGTAATTTCTACTCTTGTAGAT |
| Ef *fakB2* PAM dn | ACCTAAGCGATCATATAATTCTAATCTACAAGAGTAGAAATTATGG |
| Ef *fakB3* PAM up | agatagtggaacctataatgtggttcgAATTTCTACTCTTGTAGAT |
| Ef *fakB3* PAM dn | cgaaccacattataggttccactATCTACAAGAGTAGAAATTATGG |
| Ef *fakB4* PAM up | agattaaaacagcggcagcacttggtgAATTTCTACTCTTGTAGAT |
| Ef *fakB4* PAM dn | caccaagtgctgccgctgttttaATCTACAAGAGTAGAAATTATGG |
| pUC19U up | TAAAAAGATGCCAGTGTGCTGGAATTCGTCGATGCCAGTGTGCTGGAATT |
| pUC19U dn | TGCCAAGCTTGCATGTCTGCAGGCCTCGAGCTTGCATGTCTGCAGGCC |
| pJC005 up | CTCGAGGCCTGCAGACAT |
| pJC005 dn | GACGAATTCCAGCACACTGG |
| pBM02(*aguR*) up | GAATTCGTAATCATGTCATAGCTGTTT |
| pTRK L2(*aguR*) up | GAAAAACAACAAGCAAACTAA |
| *aguR* dn | GATGTGTTCCTCCTAAAAGT |
| Ef *fakB1* *aguR* up | AAACAACTTTTAGGAGGAACACATCATGAAAATTGCTATTGTGACAGA |
| Ef *fakB1* *aguR* dn | AAACAGCTATGACATGATTACGAATTCTTATTGAGCTGAAATCGCTAA |
| Ef *fakB2* *aguR* up | AAACAACTTTTAGGAGGAACACATCATGACAAACGTTAAAATCGTAA |
| Ef *fakB2* *aguR* dn | AAACAGCTATGACATGATTACGAATTCTTAGTCTGTATAGTACATAATAGC |
| Ef *fakB3* *aguR* up | AAACAACTTTTAGGAGGAACACATCATGAATAAAGAAAAGATCGCACTA |
| Ef *fakB3* *aguR* dn | AAACAGCTATGACATGATTACGAATTCTTAATCATTTAATAACTGTATACCAAC |
| Ef *fakB4* *aguR* up | AAACAACTTTTAGGAGGAACACATCATGAATTATCAATTAGTGACAGA |
| Ef *fakB4* *aguR* dn | AAACAGCTATGACATGATTACGAATTCTTATTTTCTTTTTTCACCAAAAGA |
| Ef *fakB1 aguR* L2 dn | ttagtttgcttgttgtttttcTTATTGAGCTGAAATCGCTAA |
| Ef *fakB2 aguR* L2 dn | ttagtttgcttgttgtttttcTTAGTCTGTATAGTACATAATAGC |
| Ef *fakB3 aguR* L2 dn | ttagtttgcttgttgtttttcTTAATCATTTAATAACTGTATACCAAC |
| Ef *fakB4 aguR* L2 dn | ttagtttgcttgttgtttttcTTATTTTCTTTTTTCACCAAAAGA |
| Ef *fabT aguR* up | AAACAACTTTTAGGAGGAACACATCATGGTGCACAGAATGG |
| Ef *fabT aguR* dn | AAACAGCTATGACATGATTACGAATTCTTATTTGTATTCTTGCAAGAAATCA |
| RT *fakA* up | TGCTAAACGTGCCTTAGCGA |
| RT *fakA* dn | CGTGACCACTAACACCACGA |
| RT *fakB1* up | TCCGACGACTTCACAACCTG |
| RT *fakB1* dn | ACCATGTGTCCCATTGGCAT |
| RT *fakB2* up | TGGACGAATCAGCCGTACAA |
| RT *fakB2* dn | AGCCCATCCGCATGAGAAAT |
| RT *fakB3* up | GGCGATACGAGCAGCAGAAT |
| RT *fakB3* dn | GCGAGACTTAGCCACCGTAT |
| RT *fakB4* up | CATTTAGAACGTGGCGGACG |
| RT 16S up | TGG TTT AAT TCG AAG CAA CG |
| RT 16S dn | CAC CAC CTG TCA CTT TGT CC |
| P32 PstI F | AAAATTCTGCAGAGATTAATAGTTTTAGCTATTAATCTTTT |
| P32 R | ATATTTTTTTACCTACCTAGTATAGCA |
| EffakB1 RBS F | CTAGGTAGGTAAAAAAATATTTAAAATATCCTTTTTTATGCTATATTAGA |
| EffakB1 5’ SalI R | ACGCGTCGACTAAGCTGTACTATCTGTCACA |
| EffakB2 RBS F | CTAGGTAGGTAAAAAAATATAATTAACCGTTTAATGGTATTCTA |
| EffakB2 5’ SalI R | ACGCGTCGACCATGAAGAATCCGTTACGA |
| EffakB3 RBS F | CTAGGTAGGTAAAAAAATATAATGTTTAGTGAAAAAGTGATTGA |
| EffakB3 5’ SalI R | ACGCGTCGACGAATCAACCAGTAGTGCG |
